# Pre-exposure Prophylaxis Use by Breastfeeding HIV-Uninfected Women: A Prospective Short-Term Study of Antiretroviral Excretion in Breast Milk and Infant Absorption

**DOI:** 10.1371/journal.pmed.1002132

**Published:** 2016-09-27

**Authors:** Kenneth K. Mugwanya, Craig W. Hendrix, Nelly R. Mugo, Mark Marzinke, Elly T. Katabira, Kenneth Ngure, Nulu B. Semiyaga, Grace John-Stewart, Timothy R. Muwonge, Gabriel Muthuri, Andy Stergachis, Connie L. Celum, Jared M. Baeten

**Affiliations:** 1 Department of Epidemiology, University of Washington, Seattle, Washington, United States of America; 2 Department of Global Health, University of Washington, Seattle, Washington, United States of America; 3 Division of Disease Control, School of Public Health, Makerere University, Kampala, Uganda; 4 Department of Medicine, Johns Hopkins University, Baltimore, Maryland, United States of America; 5 Kenya Medical Research Institute, Nairobi, Kenya; 6 Department of Medicine, Makerere University, Kampala, Uganda; 7 Jomo Kenyatta University of Agriculture and Technology, Nairobi, Kenya; 8 Infectious Diseases Institute, Makerere University, Kampala, Uganda; 9 Department of Medicine, University of Washington, Seattle, Washington, United States of America; 10 Department of Pediatrics, University of Washington, Seattle, Washington, United States of America; 11 Partners in Health Research and Development, Thika, Kenya; 12 Department of Pharmacy, University of Washington, Seattle, Washington, United States of America; Elizabeth Glaser Pediatric AIDS Foundation, UNITED STATES

## Abstract

**Background:**

As pre-exposure prophylaxis (PrEP) becomes more widely used in heterosexual populations, an important consideration is its safety in infants who are breastfed by women taking PrEP. We investigated whether tenofovir and emtricitabine are excreted into breast milk and then absorbed by the breastfeeding infant in clinically significant concentrations when used as PrEP by lactating women.

**Methods and Findings:**

We conducted a prospective short-term, open-label study of daily oral emtricitabine–tenofovir disoproxil fumarate PrEP among 50 HIV-uninfected breastfeeding African mother–infant pairs between 1–24 wk postpartum (ClinicalTrials.gov Identifier: NCT02776748). The primary goal was to quantify the steady-state concentrations of tenofovir and emtricitabine in infant plasma ingested via breastfeeding. PrEP was administered to women through daily directly observed therapy (DOT) for ten consecutive days and then discontinued thereafter. Non-fasting peak and trough samples of maternal plasma and breast milk were obtained at drug concentration steady states on days 7 and 10, and a single infant plasma sample was obtained on day 7. Peak blood and breast milk samples were obtained 1–2 h after the maternal DOT PrEP dose, while maternal trough samples were obtained at the end of the PrEP dosing interval (i.e., 23 to 24 h) after maternal DOT PrEP dose. Tenofovir and emtricitabine concentrations were quantified using liquid chromatography-tandem mass spectrometry (LC-MS/MS) assays.

Of the 50 mother–infant pairs enrolled, 48% were ≤12 wk and 52% were 13–24 wk postpartum, and median maternal age was 25 y (interquartile range [IQR] 22–28). During study follow-up, the median (IQR) daily reported frequency of infant breastfeeding was 15 times (12 to 18) overall, 16 (14 to 19) for the ≤12 weeks, and 14 (12 to 17) for the 13–24 wk infant age groups. Overall, median (IQR) time-averaged peak concentrations in breast milk were 3.2 ng/mL (2.3 to 4.7) for tenofovir and 212.5 ng/mL (140.0 to 405.0) for emtricitabine. Similarly, median (IQR) time-averaged trough concentrations in breast milk were 3.3 ng/mL (2.3 to 4.4) for tenofovir and 183.0 ng/mL (113.0 to 250.0) for emtricitabine, reflecting trough-to-peak breast milk concentration ratios of 1.0 for tenofovir and 0.8 for emtricitabine, respectively. In infant plasma, tenofovir was unquantifiable in 46/49 samples (94%), but emtricitabine was detectable in 47/49 (96%) (median [IQR] concentration: 13.2 ng/mL [9.3 to 16.7]). The estimated equivalent doses an infant would ingest daily from breastfeeding were 0.47 μg/kg (IQR 0.35 to 0.71) for tenofovir and 31.9 μg/kg (IQR 21.0 to 60.8) for emtricitabine, translating into a <0.01% and 0.5% relative dose when compared to the 6 mg/kg dose that is proposed for therapeutic treatment of infant HIV infection and for prevention of infant postnatal HIV infection; a dose that has not shown safety concerns. No serious adverse effects were recorded during study follow-up.

The key study limitation was that only a single infant sample was collected to minimize venipunctures for the children. However, maternal daily DOT and specimen collection at drug concentration steady state provided an adequate approach to address the key research question. Importantly, there was minimal variation in breast milk concentrations of tenofovir and emtricitabine (respective median trough-to-peak concentration ratio ~1), demonstrating that infants were exposed to consistent drug dosing via breast milk.

**Conclusion:**

In this short-term study of daily directly observed oral PrEP in HIV-uninfected breastfeeding women, the estimated infant doses from breast milk and resultant infant plasma concentrations for tenofovir and emtricitabine were 12,500 and >200-fold lower than the respective proposed infant therapeutic doses, and tenofovir was not detected in 94% of infant plasma samples. These data suggest that PrEP can be safely used during breastfeeding with minimal infant drug exposure.

**Trial Registration:**

ClinicalTrials.gov, Identifier: NCT02776748

## Abstract 2

In a prospective study, Kenneth Mugwanya and colleagues study the transmission of drugs used for HIV pre-exposure prophylaxis from breastfeeding mothers to their babies.

## Author Summary

**Why Was This Study Done?:**

- Pregnancy and breastfeeding represent a period of heightened HIV risk for women and for their infant if acute HIV infection occurs. Preventing HIV acquisition in women is a priority.
- Daily oral PrEP is a potent prevention option to reduce the risk of sexual HIV acquisition for women who are pregnant or breastfeeding, with the advantage relative to other prevention methods that it can be used discreetly and independent of sexual partners.
- As HIV PrEP becomes more common worldwide, large numbers of women of child-bearing age will be using these medications during pregnancy and breastfeeding.
- In order to appropriately evaluate the risk–benefit ratio of administering medications to breastfeeding women, evidence regarding infant drug exposure through breast milk is critical and very often completely lacking.

**What Did the Researchers Do and Find?:**

- We conducted an open-label, short-term, pharmacokinetic study among 50 HIV-uninfected breastfeeding women and infants.
- Women were administered directly observed PrEP, and blood and breast milk were collected to measure the concentrations of PrEP medications (i.e., tenofovir and emtricitabine); no drug was administered directly to infants.
- A single blood sample was obtained from the infant to determine the extent to which infants breastfed by women using PrEP absorb these medications via breast milk.
- We found that the concentrations of PrEP medications in infant blood were very low, and tenofovir was undetectable in 94% of the infant samples.
- The estimated doses of tenofovir and emtricitabine a breastfeeding infant would ingest each day were <0.01% and 0.5%, respectively, compared to the proposed pediatric doses for treatment of infant HIV infection and for prevention of infant postnatal HIV infection. No significant safety concerns were noted in mothers or infants.

**What Do These Findings Mean?:**

- The findings of this study provide important empirical evidence to anticipate safety for the breastfeeding infant after administering PrEP to breastfeeding HIV-uninfected women.
- These findings suggest that PrEP can be safely used during breastfeeding with minimal infant exposure and will guide practice and policy decision-making for PrEP implementation programs in heterosexual populations.
- After the pharmacokinetic demonstration of minimal drug exposure in infants breastfed by women using PrEP, the next incremental step is to explore in implementation science studies the acceptability, adherence, and infant safety in women who choose to continue PrEP throughout their pregnancy or breastfeeding and investigate long-term effects of this low-concentration exposure.

## Introduction

Women in Africa are disproportionately affected by HIV, with the greatest rates of new HIV infections among women of child-bearing age [1]. Pregnancy and the early postpartum period are characterized by heightened HIV risk associated with up to 2-fold increased HIV acquisition risk [2–5]. Moreover, vertical HIV transmission to the breastfeeding infant is a potential serious consequence of maternal acute HIV seroconversion [6]. Antiretroviral pre-exposure prophylaxis (PrEP) with emtricitabine (FTC)-tenofovir disoproxil fumarate (TDF) co-formulation or TDF alone is a highly effective strategy to reduce the risk of sexual acquisition of HIV [7–12]. The recent approval of FTC-TDF PrEP by some regulatory authorities in Africa will accelerate PrEP rollout in this region [13,14].

With expanded access to PrEP, women who are breastfeeding may be prescribed PrEP. However, only limited data are available to assess the safety of PrEP use during breastfeeding. Currently, the United States Centers for Disease Control and Prevention guidelines for PrEP permit preconception use of PrEP after discussion of the risk-benefit balance involved, but have identified the need for additional data on infant drug exposure and safety during maternal FTC-TDF PrEP use during pregnancy and postpartum breastfeeding [15,16]. We investigated whether tenofovir and emtricitabine are excreted into human milk and then absorbed by the breastfeeding infant in clinically significant concentrations in breast milk when the drugs are taken as PrEP by the lactating HIV-uninfected mother.

## Methods

### Ethics Statement

The study protocol was approved by the University of Washington Human Subjects Review Committee, the Uganda National Council of Science and Technology, the Uganda National Drug Authority, the Kenya Medical Research Institute Scientific and Ethics Unit, and the Kenyan Pharmacy and Poisons Board. All women provided written informed consent and the fathers of infants provided written permission to enroll the infant.

### Population and Study Design

This was a prospective, open-label, single-arm study of daily oral FTC-TDF PrEP among 50 HIV-uninfected lactating women and their breastfeeding infants, conducted between January and June 2015 at two clinical research sites in Thika, Kenya and Kampala, Uganda (ClinicalTrials.gov Identifier: NCT02776748). Eligible mothers were HIV seronegative and breastfeeding a singleton healthy infant, of legal age to provide written informed consent, had adequate renal function defined by normal creatinine levels and estimated creatinine clearance ≥60 mL/min, and were not infected with hepatitis B virus. Eligible infants were HIV-uninfected, aged 1–24 wk, born at term to eligible women, and had no serious infections or active clinically significant medical problems. Recruitment into the study was stratified by infant age, with half ≤12 and half 13 to 24 wk, to allow assessment of PrEP pharmacokinetics in breast milk among newborns and infants ages 3–6 mo.

### Study Procedures

Consenting HIV-uninfected breastfeeding women were followed with daily directly observed therapy (DOT) oral FTC-TDF PrEP administered at the study clinic for ten consecutive days. The ten-day schedule was chosen to attain drug concentration at steady-state levels (estimated to be achieved after five half-lives) sufficient to address the research question while minimizing potential undue infant drug exposure. PrEP was discontinued thereafter, and no medication was administered directly to infants. Co-formulated FTC and TDF were dosed at 200 mg daily and 300 mg daily, respectively; these doses are the standard doses for prevention and treatment of HIV infection. Maternal blood and breast milk samples were obtained concurrently (i.e., within 30 min of each other) regardless of the timing of food intake (i.e., non-fasting) on the seventh and tenth days. Peak samples were obtained 1–2 h after administration of maternal DOT PrEP, and trough samples were obtained at the end of the dosing interval (i.e., 23 to 24 h after DOT PrEP dose). A single infant blood sample was obtained after the seventh maternal DOT PrEP dose. All collected blood samples were centrifuged immediately at 2,000 relative centrifugal force for 15 min at room temperature, and blood plasma was aliquoted into 1 ml cryovials immediately. Breast milk was obtained by manual expression by the women and aliquoted into 1 ml cryovials immediately. All blood and breast milk specimens were stored below -80°C until testing. During daily follow-up, mothers completed a short quantitative interview about infant well-being, breastfeeding patterns, adverse events, and concomitant medication use. Both the mother and infant were monitored for adverse effects, and the severity of clinical symptoms was scored using the Division of AIDS Table for Grading the Severity of Adult and Pediatric Adverse Events [17].

### Laboratory Analytic Methods

Tenofovir and emtricitabine concentrations in plasma were quantified via previously validated liquid chromatographic-tandem mass spectrometric (LC-MS/MS) methods at the Clinical Pharmacology Analytical Laboratory at the Johns Hopkins University School of Medicine. Furthermore, LC-MS/MS methods for tenofovir and emtricitabine quantification in whole breast milk were developed and validated in accordance with the recommendations included in the US Food and Drug Administration Guidance for Industry: Bioanalytical Method Validation guidelines [18]. Briefly, tenofovir and emtricitabine were isolated from whole breast milk via protein precipitation and quantified from a blood plasma calibration curve. The lower limits of quantification for tenofovir in plasma and breast milk were 0.31 ng/mL and 1 ng/mL, respectively; and for emtricitabine in plasma and breast milk were 0.31 ng/mL and 5 ng/mL, respectively.

### Quantification of Infant Drug Exposure

The primary measure of infant drug exposure through maternal breast milk was the concentrations of tenofovir and emtricitabine in infant plasma. Secondary measures were (1) maternal plasma and whole breast milk tenofovir and emtricitabine concentrations; (2) milk to maternal plasma concentration ratios (M/P); and (3) infant plasma drug-to-milk concentration ratio. To contextualize the clinical significance of the measured drug concentrations, we estimated two additional infant indices. First, we calculated the infant drug dose received from breast milk per day (infant dose), computed as the product of breast milk tenofovir and emtricitabine concentrations and the estimated volume of breast milk consumed by the infant daily. Daily amount of breast milk consumed by the infant was assumed to be 150 mL/kg/day, the standardized milk consumption of the mean milk intake of a fully breastfed infant [19,20]. Second, we calculated infant dose fraction, which is the drug dose a fully breastfed infant would ingest from maternal milk as a fraction of the infant’s weight-adjusted therapeutic dose. This was computed from infant daily dose from breast milk and the weight-adjusted recommended therapeutic pediatric doses as infant dose fraction (%) = infant dose from breast milk*100/infant therapeutic dose [20,21]. The respective therapeutic doses for tenofovir and emtricitabine were considered to be 6 mg/kg; these doses were derived from published doses considered be effective for prevention of mother to child transmission of HIV for age equivalent populations and have not shown safety concerns [22–25]. All outcome measures were evaluated separately for tenofovir and emtricitabine concentrations and stratified by the timing of sample collection (i.e., trough or peak).

### Statistical Analysis

The primary outcome was the proportion of infants with detectable steady-state concentrations of tenofovir and emtricitabine in plasma, overall, and stratified by infant age (i.e., ≤12 wk or 13–24 wk). Data were summarized as medians and interquartile ranges (IQR) for continuous variables and proportions for categorical outcomes. For one peak maternal plasma record, the tenofovir concentration (1,040.0 ng/mL) was out of the assay analytic range (0.31–1,000.0 ng/mL). This record was imputed to the upper limit of the assay analytic range. For concentrations below the assay limit of detection, a value of one-half of the detection limit was used in summary calculations for continuous variables; where >3 samples are below the lower of limit of quantification, the proportion of samples with undetectable levels are instead presented. Mann–Whitney *U* test was used to compare the distribution of infant peak concentrations, daily dose from milk, and drug exposure index between the two infant age strata. All analyses were conducted in SAS version 9.4, SAS Institute Inc., Cary, North Carolina, US.

## Results

### Population Characteristics and Follow-Up

Of the 50 mother–infant pairs enrolled, 24 (48%) infants were ≤12 weeks of age, and median (IQR) weight at study entry was 5 kg (4.3–6.0) for the ≤12 wk group and 6.6 kg (6.0–7.1) for the 13–24 wk group. Infants were breastfed for median of 15 (IQR 12–18) times daily during the week prior to study participation (Table 1); median daily proportion of infant food intake derived from breastfeeding in the week prior to study entry was 100%. During study follow-up, the median (IQR) daily frequency of infant breastfeeding was 15 times (12–18) overall, 16 (14–19) for the ≤12 wk and 14 (12–17) for the 13–24 wk infant age groups.

**Table 1:** General characteristics.

Characteristic	All infants (*n* = 50)	Infant age ≤12 wk (*n* = 24)	Infant age 13–24 weeks (*n* = 26)
Infant age in weeks	13 (9–19)	9 (6–10)	19 (17–21)
Birth weight in kg	3.4 (3.0–3.5)	3.3 (3.0–3.7)	3.4 (2.8–3.5)
Infant weight at screening in kg	6.0 (5.0–6.7)	5 (4.3–6.0)	6.6 (6.0–7.1)
Maternal age in years	25 (22–28)	24 (22–28)	26 (22–28)
Infant length in cm	58 (55–61)	55 (52–58)	60 (58–62)
Average daily frequency of breastfeeding, past week	15 (12–18)	16 (8–25)	15 (6–20)
Median proportion of infant feed due to breastfeeding	100% (100–100)	100% (100–100)	100% (100–100)
Maternal creatinine clearance in mL/min	107 (93–120)	109 (95–120)	105 (93–119)
Maternal serum creatinine in mg/dL	0.64 (0.58–0.71)	0.60 (0.57–0.68)	0.66 (0.58–0.72)
Maternal AST	21 (19–24)	22 (19–24)	20 (19–24)
Maternal ALT	19 (14–23)	19 (14–23)	22 (15–27)

Statistics are median (interquartile range) for continuous covariates and percent for binary variables. ALT, Alanine transaminase; AST, Aspartate aminotransferase.

Overall, 499 of 500 (>99%) daily DOT PrEP doses were taken by the mother, and 439 of 450 (98%) expected samples for pharmacokinetic analysis were collected: 195 maternal plasma (98 for peak and 97 for trough); 195 breast milk (98 for peak and 97 for trough); and 49 infant plasma samples. Peak maternal blood, breast milk, and infant blood samples were obtained after a median (IQR) of 63 (60 to 68), 70 (65 to 77), and 80 (45 to 90) min after maternal DOT PrEP dose, respectively, whereas maternal trough samples were obtained a median of 23 h (IQR 23 to 24) from the previous maternal DOT PrEP dose.

### Tenofovir and Emtricitabine Concentrations in Maternal Plasma and Breast Milk

**Fig 1:**
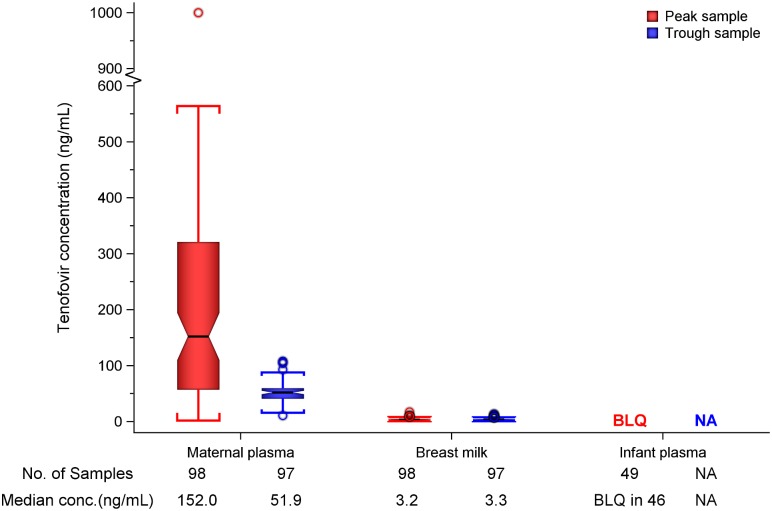
Box plot of maternal and infant tenofovir concentrations. Non-fasting maternal blood and breast milk samples were obtained concurrently (i.e., within 30 min) at the seventh and tenth visits (corresponding to seventh and tenth maternal DOT PrEP doses). A single infant blood sample was obtained after the seventh maternal DOT PrEP dose. Peak maternal blood, breast milk, and infant blood samples were obtained a median (IQR) of 63 (60 to 68), 70 (65 to 77), and 80 (45 to 90) min after the maternal DOT PrEP dose, respectively. Trough samples were obtained at close of the dosing interval, a median of 23 h (IQR range 23 to 24) after the previous maternal DOT PrEP. One outlier peak maternal plasma tenofovir concentration (1,040 ng/ml) was out of the assay analytic range (0.31–1,000.0 ng/mL). This record was imputed to the upper limit of the assay analytic range and was included in the computation of the displayed summary estimate. Middle box line represents the median. Upper box line represents the 75th percentile and the lower box line represents the 25th percentile. The top whisker denotes the maximum data value or the third quartile plus 1.5 times the interquartile range, whichever is smaller. The lower whisker denotes the minimum data value or the third quartile plus 1.5 times the interquartile range, whichever is larger. The notches display the 95% confidence interval around the median. Small circles represent outlier data points (i.e., observations that are as extreme as ±1.5 of interquartile range). Only 3 of 49 infant plasma samples had quantifiable tenofovir concentration in plasma (infants aged 11 and 13 wk [both 0.9 ng/mL] and 17 wks [17.4 ng/mL]). NA, not applicable; BLQ, below assay limit of quantification for tenofovir: <0.31 ng/mL in plasma and <1 ng/mL in whole milk.

In maternal plasma, tenofovir was detected at concentrations consistent with steady-state use, and breast milk tenofovir concentrations were considerably lower than those in maternal plasma (Fig 1). The median (IQR) time-averaged peak steady-state concentrations of tenofovir in maternal plasma and breast milk were 152.0 ng/mL (IQR 56.9 to 321.0) and 3.2 ng/mL (2.3 to 4.7), respectively, resulting in a median peak milk/plasma (M/P) ratio of 0.03 (0.01 to 0.05) (Table 2). Similarly, median (IQR) time-averaged trough steady-state concentrations of tenofovir in maternal plasma and breast milk were 51.9 ng/mL (IQR 40.7 to 59.6) and 3.3 ng/mL (2.3 to 4.4), respectively, representing a trough median M/P ratio of 0.07 (IQR 0.05 to 0.08).

**Table 2:** Tenofovir concentrations and infant exposure.

Variable	All infants	Infant age ≤12 wk	Infant age 13–24 wk	*p*-value
***Peak*** *	***n* = 98**	***n* = 49**	***n* = 49**	
Maternal plasma concentration in ng/mL	152.0 (56.9–321.0)	140.5 (53.3–327.5)	165.5 (58.4–309.0)	
Breast milk concentration in ng/mL	3.2 (2.3–4.7)	3.8 (2.7–6.9)	2.9 (2.1–3.8)	
M/P concentration ratio	0.03 (0.01–0.05)	0.03 (0.02–0.07)	0.02 (0.01–0.04)	
Proportion of infant plasma samples with concentration below the lower limit of quantification†	94% (46/49)	96% (23/24)	92% (24/25)	
Infant daily dose from breast milk in μg/kg	0.47 (0.35–0.71)	0.57 (0.41–1.04)	0.44 (0.32–0.56)	0.06
Infant dose fractionⱡ	<0.01% (<0.01–0.01)	<0.01% (0–0.02)	<0.01% (<0.01–0.01)	0.06
***Trough*** *	***n* = 97**	***n* = 48**	***n* = 49**	
Maternal plasma concentration in ng/mL	51.9 (40.7–59.6)	54.1 (45.7–62.3)	46.0 (39.4–57.2)	
Breast milk concentration in ng/mL	3.3 (2.3–4.4)	3.5 (2.3–6.8)	3.2 (2.3–3.8)	
M/P concentration ratio	0.07 (0.05–0.08)	0.07 (<0.01–0.31)	0.07 (<0.01–0.11)	
Infant daily dose from breast milk in μg/kg	0.49 (0.34–0.66)	0.52 (0.05–0.08)	0.49 (0.05–0.08)	0.11
Infant dose fractionⱡ	<0.01% (<0.01–0.01)	<0.01% (<0.01–0.01)	<0.01% (<0.01–0.01)	0.11

Unless stated, statistics are median (interquartile range). *n* are for samples tested, with each woman providing a maximum of two of respective records (i.e, one on day 7 and another on day 10), while each infant provided one record.

*Peak maternal blood, breast milk, and infant blood samples were obtained after a median (IQR) of 63 (60 to 68), 70 (65 to 77), and 80 (45 to 90) min after maternal DOT PrEP dose, respectively, while maternal trough samples were obtained a median of 23 h (IQR 23 to 24) from the previous maternal DOT PrEP dose.

^†^ *n* = 49, a single infant plasma sample was obtained: 24 samples for ≤12 wk age group and 25 samples for 13–24 wk age group. Only 3 of 49 infant plasma samples had detectable tenofovir concentration in plasma (Infants aged 11 and 13 wk [both had 0.9 ng/mL] and 17 wk [17.4 ng/mL]).

^ⱡ^ Infant dose fraction, represents the daily amount of tenofovir dose an infant would be expected to ingest from breast milk as a percentage of the proposed therapeutic daily dose (6 mg/kg).

*p*-Values are from Mann–Whitney *U* test testing the null hypothesis that the two infant age groups are drawn from the same distribution.

M/P, milk to maternal plasma ratio; Lower limit of quantification was <0.31 ng/mL in plasma and <1 ng/mL in whole breast milk.

**Fig 2:**
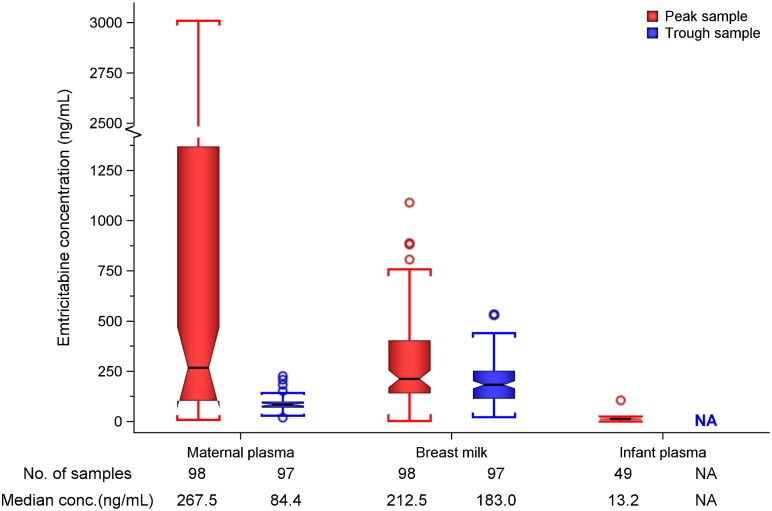
Box plot of maternal and infant emtricitabine concentrations. Non-fasting maternal blood and breast milk samples were obtained concurrently (i.e., within 30 min) at the seventh and tenth visits (corresponding to seventh and tenth maternal DOT PrEP doses). A single infant blood sample was obtained after the seventh maternal DOT PrEP dose. Peak maternal blood, breast milk, and infant blood samples were obtained a median (IQR) of 63 (60 to 68), 70 (65 to 77), and 80 (45 to 90) min after the maternal DOT PrEP dose, respectively. Trough samples were obtained at close of the dosing interval, a median of 23 h (IQR range 23 to 24) after the previous maternal DOT PrEP. Middle box line represents the median. Upper box line represents the 75th percentile, and the lower box line represents the 25th percentile. The top whisker denotes the maximum data value or the third quartile plus 1.5 times the interquartile range, whichever is smaller. The lower whisker denotes the minimum data value or the third quartile plus 1.5 times the interquartile range, whichever is larger. The notches display the 95% confidence interval around the median. Small circles represent outlier data points (i.e., observations that are as extreme as ±1.5 of interquartile range). NA, not applicable; BLQ, below assay limit of quantification for emtricitabine: <0.31 ng/mL in plasma and <5 ng/mL in whole milk.

Maternal plasma emtricitabine concentrations were also consistent with steady-state use, and emtricitabine concentrations in breast milk were more similar to plasma concentrations than had been seen for tenofovir (Fig 2). The median (IQR) time-averaged peak steady-state concentrations of emtricitabine in maternal plasma and breast milk were 267.5 ng/mL (103.0 to 1370.0) and 212.5 ng/mL (140.0 to 405.0), respectively, representing a median peak M/P ratio of 0.63 (0.31 to 1.43) (Table 3). Similarly, the median (IQR) time-averaged trough steady-state concentrations of emtricitabine in maternal plasma and breast milk were 84.4 ng/mL (68.5 to 99.7) and 183.0 ng/mL (113.0 to 250.0), respectively, representing a median trough M/P ratio of 2.1 (IQR 1.67 to 2.81). Overall, in contrast to maternal plasma concentrations, there was less variability in concentration of both tenofovir and emtricitabine in breast milk (median [range] trough to peak breast milk concentration ratio; 1.0 [0.7 to 1.3] for tenofovir and 0.8 [0.4 to 1.3] for emtricitabine).

**Table 3:** Emtricitabine concentrations and infant exposure.

Variable	All infants	Infant age ≤12 wk	Infant age 13–24 wk	*p*-value
***Peak*** *	***n* = 98**	***n* = 49**	***n* = 49**	
Maternal plasma concentration in ng/mL	267.5 (103.0–1370.0)	236.5 (93.6–1380.0)	533.0 (115.0–1370.0)	
Breast milk concentration in ng/mL	212.5 (140.0–405.0)	208.0 (139.5–377.5)	215.0 (149.0–431.0)	
M/P concentration ratio	0.63 (0.31–1.43)	0.70 (0.31–1.76)	0.59 (0.31–1.14)	
Infant plasma concentrationⱡ in ng/mL	13.2 (9.3–16.7)	16.6 (13.2–20.9)	10.5 (7.1–13.2)	<0.01
Infant plasma/milk concentration ratio	0.05 (0.03–0.08)	0.07 (0.04–0.10)	0.05 (0.02–0.06)	0.12
Infant daily dose from breast milk in μg/kg	31.9 (21.0–60.8)	31.2 (20.9–56.6)	32.3 (22.4–64.7)	0.94
Infant dose fraction†	0.5% (0.3–1.0)	0.5% (0.3–0.9)	0.5% (0.4–1.1)	0.94
***Trough*** *	***n* = 97**	***n* = 48**	***n* = 49**	
Maternal plasma concentration in ng/mL	84.4 (68.5–99.7)	82.8 (69.3–101.0)	84.8 (68.2–97.5)	
Breast milk concentration in ng/mL	183.0 (113.0–250.0)	187.5 (95.6–256.0)	183.0 (125.0–250.0)	
M/P concentration ratio	2.10 (1.67–2.81)	2.36 (1.48–2.83)	2.08 (1.69–2.81)	
Infant daily dose from breast milk in μg/kg	27.5 (17.0–37.5)	28.1 (14.3–38.4)	27.5 (18.9–37.5)	0.58
Infant dose fraction†	0.5% (0.3–0.6)	0.5% (0.2–0.6)	0.5% (0.3–0.6)	0.58

Unless stated, statistics are median (interquartile range); *n* are for samples tested, with each woman providing a maximum of two of respective records (i.e, one on day 7 and another on day 10), while each infant provided one record.

*Peak maternal blood, breast milk, and infant blood samples were obtained after a median (IQR) of 63 (60 to 68), 70 (65 to 77), and 80 (45 to 90) min after maternal DOT PrEP dose, respectively, while maternal trough samples were obtained a median of 23 h (IQR 23 to 24) from the previous maternal DOT PrEP dose.

^ⱡ^ *n* = 49, a single infant plasma sample was obtained: 24 samples for ≤12 wk age group and 25 for 13–24 wk age group. Emtricitabine was unquantifiable in 2 of 49 infant plasma samples.

^†^Infant dose fraction (also called exposure index) represents the daily amount of emtricitabine dose an infant would ingest from breast milk as a percentage of the proposed pediatric therapeutic daily dose (6 mg/kg).

p-values are from Mann–Whitney *U* test testing the null hypothesis that the two infant age groups are drawn from the same distribution.

M/P milk to maternal plasma ratio; Lower limit of quantification was <0.31 ng/mL in plasma and <5 ng/mL in whole breast milk.

### Infant Exposure to Tenofovir and Emtricitabine from Maternal Breast Milk

Overall, after seven consecutive maternal daily DOT FTC-TDF PrEP, tenofovir was undetectable in 46 of 49 (94%) infant plasma samples; the three infants with detectable tenofovir also had detectable emtricitabine. These three infants were ages 11, 13, and 17 wk (plasma concentrations 0.9, 0.9, and 17.4 ng/mL, respectively, and body weight 6.4, 5.8, and 6.2 kg, respectively), and their maternal milk tenofovir concentrations were modestly greater than the overall median (6.57, 3.64, and 4.05 ng/mL, respectively, versus median 3.2 ng/mL). There were no other notable unique characteristics between these three mother–infant pairs and the others. The median amount of tenofovir dose estimated to be ingested by an infant from breast milk was 0.47 μg/kg (IQR 0.35 to 0.71), translating into <0.01% (i.e., 12,500-fold lower) of the proposed pediatric tenofovir therapeutic daily dose (6 mg/kg) [24,25]. Specifically, a 5-kg body weight infant would be expected to ingest a total tenofovir dose of 2.35 x 10^−3^ mg daily from breast milk compared to the proposed therapeutic daily dose of 30 mg.

Emtricitabine was detectable in 47 of 49 (96%) infant plasma samples. The median (IQR) emtricitabine concentration in infant plasma was 13.2 ng/mL (9.3 to 16.7) overall, approximately 5% of breast milk concentrations: 16.6 ng/mL for infants aged ≤12 wk and 10.5 ng/mL in infants 13–24 wk. Based on the steady-state concentrations, the estimated median dose of emtricitabine expected to be ingested by the infant per day from breastfeeding was 31.9 μg/kg (IQR 21.0 to 60.8), translating into 0.5% (i.e., 200-fold lower) of the proposed pediatric emtricitabine therapeutic daily dose (6 mg/kg) [22,24]; the estimated doses were similar in the two infant age groups (Table 3). Specifically, a 5-kg body weight infant would ingest from breastfeeding a total daily emtricitabine dose of 0.16 mg compared to the recommended therapeutic dose of 30 mg per day.

### Safety and Tolerance

FTC-TDF was well tolerated by study mothers and infants. Over the ten-day maternal FTC-TDF PrEP dosing period, clinical symptoms recorded on ≥2 occasions were abdominal pain, diarrhea, and nausea in three (6%), two (4%), and three (6%) women, respectively (abdominal pain and nausea were concurrent in two women). In two infants (4%), diarrhea was reported on two visits during the study duration. These symptoms in both mother and infant were mild and resolved in 2–3 d. Of 50 women, 48 completed a safety kidney function screen at exit. Calculated creatinine clearance was >90 mL/min at baseline and exit for all women (median: serum creatinine [0.64 versus 0.66 mg/dL] and creatinine clearance [107 versus 101 ml/min]).

## Discussion

In this prospective study of daily, directly observed doses of daily oral FTC-TDF PrEP in HIV-uninfected breastfeeding women, the estimated infant doses from breastfeeding and the resultant infant plasma concentrations for both tenofovir and emtricitabine were far below what would result from the proposed pediatric doses. Based on breast milk concentration measurements, the estimated daily tenofovir and emtricitabine doses ingested by the infant through breastfeeding were 12,500-fold and 200-fold, respectively, lower than the proposed daily pediatric dose for prophylaxis against vertical HIV acquisition. Thus, infants had low exposures to tenofovir and emtricitabine, which would not be expected to pose substantial safety risk to infants of mothers who use PrEP during breastfeeding.

To our knowledge, this is the first study to directly assess infant drug exposure via breast milk of mothers using FTC-TDF PrEP. We implemented an intensive daily maternal DOT PrEP dosing schedule to remove variability due to adherence. Daily oral PrEP offers an effective female-controlled option to reduce the risk of sexual HIV acquisition for women who are pregnant or breastfeeding, with the advantage relative to other prevention methods that it does not require cooperation of sexual partners. These data provide important empirical evidence to inform the discussion and assessment of risk-to-benefit balance of initiating or continuing PrEP during breastfeeding and are informative for evidence-based clinical guidelines. Although we were unable to implement a full concentration–time pharmacokinetic profile approach, our data collected at steady state demonstrate minimal variation in the concentrations of tenofovir and emtricitabine in breast milk, indicating that infants acquired consistent drug dosing throughout the day via breast milk. Thus, our findings suggest that PrEP can be safely used during breastfeeding with minimal infant exposure.

Our study provides both novel and complementary findings to the Agence Nationale de Recherche sur le Sida (ANRS) 12109 study [26], a pharmacokinetic study that assessed tenofovir and emtricitabine exposure in five HIV-infected Ivorian women dosed at 400 mg FTC–600 mg TDF at the start of labor and 200 mg FTC–300 mg TDF daily for 7 d postpartum. In the 16 breast milk samples obtained in that study, simulated peak median infant tenofovir and emtricitabine daily doses from breast milk were 4.2 μg/kg and 146 μg/kg, respectively, which represented 0.03% and 2% of the respective therapeutic oral infant doses. Notably, these estimated infant doses are larger than those we found in this study (estimated infant daily doses from breast milk: tenofovir = 0.47 μg/kg and emtricitabine = 31.9 μg/kg). One explanation is the difference in dosing and sampling schedules between the two studies. Alternatively, the difference could mean that infants are exposed to far smaller tenofovir and emtricitabine concentrations from breast milk based on direct plasma measure in our study than anticipated from the simulated doses in the ANRS study. Importantly, infant plasma drug concentrations were not directly measured in that study.

For breastfeeding women taking oral TDF, breast milk will exclusively contain tenofovir in an unesterified anionic form, and, due to its poor oral bioavailability, negligible tenofovir concentrations would be expected to be absorbed by the infant from breastfeeding, consistent with our findings. In contrast, emtricitabine, which has excellent bioavailability, would be expected be absorbed to some degree from breast milk by the infant, as has been observed with the structurally similar lamivudine and which was seen in this study. Although emtricitabine concentrations were quantified in infant plasma, the concentration we observed in this study was a small fraction (~0.5%) of the infant therapeutic daily doses used to treat HIV.

For most drugs, including tenofovir and emtricitabine, the dose below which there is no clinical effect in infants is unknown. A dose fraction index (exposure of 10% weight-adjusted therapeutic pediatric dose) has been proposed as a safety threshold for infant exposure to maternal drugs from breast milk, below which the degree of exposure to the drug in breast milk is considered clinically unimportant [27]. In this study, we found infant plasma tenofovir and emtricitabine concentrations to be only <0.01% and 0.5% of the respective proposed therapeutic pediatric doses. Accordingly, for TDF-based PrEP use during lactation, the small concentrations of tenofovir and emtricitabine absorbed by infants from maternal breast milk observed in our study are likely to be of limited clinical consequence.

Our results must be interpreted in light of the following limitations. First, we only tested for plasma tenofovir and emtricitabine concentrations, not their pharmacologically active intracellular derivatives, tenofovir-triphosphate and emtricitabine-diphosphate concentrations, respectively. Second, we collected only a single infant sample to minimize venipunctures for children. In sparse data pharmacokinetic situations like our study, in which the traditional full drug concentration–time profile approach is not applicable, daily DOT and a steady-state sampling provide an adequate approach to address our key research question. Importantly, there was minimal variation in the concentrations of tenofovir and emtricitabine in breast milk, demonstrating that infants were exposed to consistent tenofovir and emtricitabine dosing throughout the day via breast milk. Third, quantifying the volume of milk intake was not feasible, so we used the standard assumption of 150 mL/kg/day breast milk intake of a fully fed infant. Fourth, we only tested peak and trough maternal concentrations, which limits the precision of our M/P estimates throughout a dosing interval. Fifth, maternal blood and breast milk samples were obtained concurrently (i.e., within 30 min of each other). It is possible that a lag in blood-to-breast milk excretion could impact the observed breast milk concentrations. Other studies have used a similar approach when sampling breast milk, and this lag was not readily evident in a previous animal model of FTC-TDF breast milk excretion in two macaques [26,28]. Similar to our findings, minimal variations in breast milk concentrations of nucleoside/nucleotide reverse transcriptase inhibitor concentrations have previously been reported in HIV-infected breastfeeding women, potentially owing to the slow elimination from breast milk [29]. Thus, given the steady-state sampling approach in our study, any potential lag in blood-to-breast milk excretion would be expected to have minimal effects on our findings.

In conclusion, in this prospective study among HIV-uninfected breastfeeding African women using DOT FTC-TDF PrEP, nursing infants were exposed to lower tenofovir and emtricitabine concentrations from breastfeeding than the proposed pediatric therapeutic doses. These data provide evidence suggesting that this PrEP regimen can be safely used during breastfeeding, which is informative for clinical guidelines for women who are at substantial risk of HIV during pregnancy and the postpartum period.

## Supporting Information

S1 Table Data underlying Figs 1 and 2. (XLSX)

S1 Text STROBE checklist. (DOCX)

S2 Text Study protocol. (PDF)

## Abbreviations

BLQ: below limit of quantification
DOT: directly observed therapy
FTC: emtricitabine
IQR: interquartile range
LC-MS/MS: liquid chromatography-tandem mass spectrometry
M/P: maternal plasma concentration ratio
PrEP: pre-exposure prophylaxis
TDF: tenofovir disoproxil fumarate
