# Supplementary material for: Pre-exposure Prophylaxis Use by Breastfeeding HIV-Uninfected Women: A Prospective Short-Term Study of Antiretroviral Excretion in Breast Milk and Infant Absorption
Source: PLoS Med. 2016 Sep 27;13(9):e1002132. doi: 10.1371/journal.pmed.1002132 (PMC5038971; doi:10.1371/journal.pmed.1002132)
Supplement: S1 Text — (DOCX) [file pmed.1002132.s002.docx]

STROBE Statement—checklist of items that should be included in reports of observational studies

|  | Item No | Recommendation | Present manuscript |
| --- | --- | --- | --- |
| **Title and abstract** | 1 | (*a*) Indicate the study’s design with a commonly used term in the title or the abstract | **Done (“prospective short-term study” in title).** |
|  |  | (*b*) Provide in the abstract an informative and balanced summary of what was done and what was found | **Done (throughout Abstract and summarized in “Interpretation” section).** |
| Introduction | | |  |
| Background/rationale | 2 | Explain the scientific background and rationale for the investigation being reported | **Done (throughout Introduction).** |
| Objectives | 3 | State specific objectives, including any prespecified hypotheses | **“The overall goal was to investigate whether tenofovir and emtricitabine are excreted into breastmilk and then absorbed by the breastfeeding infant in clinically significant concentrations when used as PrEP by lactating women.”** |
| Methods | | |  |
| Study design | 4 | Present key elements of study design early in the paper | **Done (Methods).** |
| Setting | 5 | Describe the setting, locations, and relevant dates, including periods of recruitment, exposure, follow-up, and data collection | **Done (2^st^ paragraph of Methods section subsection: Population and study design).** |
| Participants | 6 | (*a*) *Cohort study*—Give the eligibility criteria, and the sources and methods of selection of participants. Describe methods of follow-up  *Case-control study*—Give the eligibility criteria, and the sources and methods of case ascertainment and control selection. Give the rationale for the choice of cases and controls  *Cross-sectional study*—Give the eligibility criteria, and the sources and methods of selection of participants | **Done (2^st^ paragraph of Methods section, subsection: Population and study design).** |
|  |  | (*b*) *Cohort study*—For matched studies, give matching criteria and number of exposed and unexposed  *Case-control study*—For matched studies, give matching criteria and the number of controls per case | **N/A** |
| Variables | 7 | Clearly define all outcomes, exposures, predictors, potential confounders, and effect modifiers. Give diagnostic criteria, if applicable | **“The primary measure of infant drug exposure through maternal breastmilk was the concentrations of tenofovir and emtricitabine in infant plasma. Secondary measures were: a) maternal plasma and whole breastmilk tenofovir and emtricitabine concentrations; b) milk to maternal plasma concentration ratios (M/P); and c) infant plasma drug to milk concentration ratio. To contextualize the clinical significance of the measured drug concentrations, we estimated two additional infant indices: 1) infant drug dose received from breastmilk per day (infant dose), and 2) infant dose fraction, the drug dose a fully breastfed infant would ingest from maternal milk as a fraction of the infant’s therapeutic dose per body weight.”** |
| Data sources/ measurement | 8* | For each variable of interest, give sources of data and details of methods of assessment (measurement). Describe comparability of assessment methods if there is more than one group | **As per #7.** |
| Bias | 9 | Describe any efforts to address potential sources of bias | **Detailed in Results and discussion.** |
| Study size | 10 | Explain how the study size was arrived at | **“The sample size of 50-mother-infant pairs was chosen to permit a thorough evaluation of antiretroviral excretion into breast milk and infant drug exposure via breastfeeding at drug steady states. This sample size is consistent with samples sizes used in similar antiretroviral pharmacokinetic studies.”** |
| Quantitative variables | 11 | Explain how quantitative variables were handled in the analyses. If applicable, describe which groupings were chosen and why | **Detailed in Statistical Analysis section.** |
| Statistical methods | 12 | (*a*) Describe all statistical methods, including those used to control for confounding | **Detailed in Statistical Analysis section.** |
|  |  | (*b*) Describe any methods used to examine subgroups and interactions | **Recruitment into the study was stratified by infant age, with half ≤12 and half 13 to 24 weeks, to allow assessment of PrEP pharmacokinetics in breastmilk among newborns and infants ages 3-6 months.** |
|  |  | (*c*) Explain how missing data were addressed | **For concentrations below the assay limit of detection, a value of one-half of the detection limit was used in summary calculations for continuous variables; where >3 samples are below the lower of limit of quantification, the proportion of samples with undetectable levels are instead presented** |
|  |  | (*d*) *Cohort study*—If applicable, explain how loss to follow-up was addressed  *Case-control study*—If applicable, explain how matching of cases and controls was addressed  *Cross-sectional study*—If applicable, describe analytical methods taking account of sampling strategy |  |
|  |  | (*e*) Describe any sensitivity analyses |  |

| Results | | |  |
| --- | --- | --- | --- |
| Participants | 13* | (a) Report numbers of individuals at each stage of study—eg numbers potentially eligible, examined for eligibility, confirmed eligible, included in the study, completing follow-up, and analysed | **This information is provided in the first paragraph of the Results.** |
|  |  | (b) Give reasons for non-participation at each stage |  |
|  |  | (c) Consider use of a flow diagram |  |
| Descriptive data | 14* | (a) Give characteristics of study participants (eg demographic, clinical, social) and information on exposures and potential confounders | **This information is provided in the first paragraph of the Results and in Table 1.** |
|  |  | (b) Indicate number of participants with missing data for each variable of interest | **Table 1.** |
|  |  | (c) *Cohort study*—Summarise follow-up time (eg, average and total amount) | **“50 mother-infant pair were follow-d 10 day consecutive days. All pairs completed follow up.** |
| Outcome data | 15* | *Cohort study*—Report numbers of outcome events or summary measures over time | **“Tenofovir was undetectable in 46 of 49 (94%) infant plasma samples. Emtricitabine was detectable in 47 of 49 (96%) infant plasma samples. The median amount of tenofovir dose estimated to be ingested by an infant from breastmilk was 0.47 µg/kg (IQR 0.35 to 0.71), translating into <0.01% (i.e. 12500-fold lower) of the proposed pediatric tenofovir therapeutic daily dose (6 mg/kg) The estimated median dose of emtricitabine expected to be ingested by the infant per day from breastfeeding was 31.9 µg/kg (IQR 21.0 to 60.8), translating into 0.5% (i.e. 200-fold lower) of the proposed pediatric emtricitabine therapeutic daily dose (6 mg/kg)** |
|  |  | *Case-control study—*Report numbers in each exposure category, or summary measures of exposure |  |
|  |  | *Cross-sectional study—*Report numbers of outcome events or summary measures |  |
| Main results | 16 | (*a*) Give unadjusted estimates and, if applicable, confounder-adjusted estimates and their precision (eg, 95% confidence interval). Make clear which confounders were adjusted for and why they were included | **“Median and interquartile ranges provided through text and in Table 2 and 3. Summary measures stratified on infant age to reflect changes in breast milk composition over varying postpartum weeks as well as changes in infant feeding, absorption, and clearance patterns -factors which contribute to the time- and phase-dependent variation of drug excretion into milk and subsequent infant absorption.”** |
|  |  | (*b*) Report category boundaries when continuous variables were categorized | **Table 2 and 3** |
|  |  | (*c*) If relevant, consider translating estimates of relative risk into absolute risk for a meaningful time period | **NA** |
| Other analyses | 17 | Report other analyses done—eg analyses of subgroups and interactions, and sensitivity analyses | **Infant age- subgroups in Table 2 and 3.** |
| Discussion | | |  |
| Key results | 18 | Summarise key results with reference to study objectives | **First paragraph of Discussion.** |
| Limitations | 19 | Discuss limitations of the study, taking into account sources of potential bias or imprecision. Discuss both direction and magnitude of any potential bias | **Second to last paragraph of Discussion.** |
| Interpretation | 20 | Give a cautious overall interpretation of results considering objectives, limitations, multiplicity of analyses, results from similar studies, and other relevant evidence | **Done.** |
| Generalisability | 21 | Discuss the generalisability (external validity) of the study results | **“The liquid chromatographic-tandem mass spectrometric methods for tenofovir and emtricitabine quantification in whole breastmilk were developed and validated in accordance with the recommendations included in the US Food and Drug Administration, Guidance for Industry, Bioanalytical Method Validation guidelines. It is possible that potential variability in drug disposition due to genetic polymorphisms in drug disposition genes (CYP2B6, NR1I3, CYP2A6, ABCB1, ABCB5, and ABCG2) may differ between African populations compared to Caucasians. However, the prevalence of these polymorphisms is unknown in both populations and their contribution is likely to be of limited clinical significance at a population level.** |
| Other information | | |  |
| Funding | 22 | Give the source of funding and the role of the funders for the present study and, if applicable, for the original study on which the present article is based | **“This work was supported by the Bill & Melinda Gates Foundation (OPP47674). The funders had no role in study design, data collection and analysis, decision to publish, or preparation of the manuscript. Gilead Sciences donated the PrEP medication but had no role in data collection or analysis. The results and interpretation presented here do not necessarily reflect the views of the study funders.”** |

*Give information separately for cases and controls in case-control studies and, if applicable, for exposed and unexposed groups in cohort and cross-sectional studies.

**Note:** An Explanation and Elaboration article discusses each checklist item and gives methodological background and published examples of transparent reporting. The STROBE checklist is best used in conjunction with this article (freely available on the Web sites of PLoS Medicine at http://www.plosmedicine.org/, Annals of Internal Medicine at http://www.annals.org/, and Epidemiology at http://www.epidem.com/). Information on the STROBE Initiative is available at www.strobe-statement.org.
