# Supplementary material for: Pre-exposure Prophylaxis Use by Breastfeeding HIV-Uninfected Women: A Prospective Short-Term Study of Antiretroviral Excretion in Breast Milk and Infant Absorption
Source: PLoS Med. 2016 Sep 27;13(9):e1002132. doi: 10.1371/journal.pmed.1002132 (PMC5038971; doi:10.1371/journal.pmed.1002132)
Supplement: S2 Text — (PDF) [file pmed.1002132.s003.pdf]

PROTOCOL

An open-label, short-duration, repeat-dose study of breast milk excretion and infant absorption of daily oral tenofovir disoproxil fumarate/emtricitabine when used by HIV-1 uninfected lactating women

Version 1.0

05 August, 2014

Sponsor:
University of Washington

Funding:
Bill & Melinda Gates Foundation

STUDY TEAM

University of Washington

Jared Baeten, MD, PhD (Principal Investigator)	University of Washington, Seattle, USA
Connie Celum, MD, MPH	University of Washington, Seattle, USA
Kenneth Mugwanya, MBChB, MS	University of Washington, Seattle, USA

Johns Hopkins University

Craig Hendrix, MD	Johns Hopkins University, Baltimore, USA

Kampala, Uganda site

Elly Katabira, MBChB	Makerere University, Kampala, Uganda

Thika, Kenya site

Nelly Mugo, MBChB, MMed, MPH	Kenya Medical Research Institute, Nairobi, Kenya
Kenneth Ngure, MPH, PhD	Jomo Kenyatta University of Agriculture and Technology, Nairobi, Kenya

I. Summary

Pre-exposure prophylaxis (PrEP) with tenofovir disoproxil fumarate (TDF) alone or in combination with emtricitabine (FTC/TDF) reduces the risk of HIV-1 acquisition in high-risk HIV-1-uninfected adults.[1-4] Clinical trials have demonstrated protection efficacies of 44-75% in randomized comparisons to placebo and ~90% in case-control analyses of participants with high PrEP adherence. On the basis of these data, the US Food and Drug Administration (FDA) approved the use of FTC/TDF for PrEP in July 2012[5]. The World Health Organization also issued guidance on oral PrEP, specifically for heterosexual HIV-1 serodiscordant couples and men and transgender women who have sex with men, regarding use in the context of demonstration projects to garner additional information to facilitate policies about PrEP implementation[6]. In addition, the US Centers for Disease Control and Prevention (CDC) issued interim guidance for clinicians considering using PrEP for prevention of HIV-1 infection in high-risk heterosexually active adults[7], and identified the need for data on infant safety during pregnancy and postpartum breastfeeding for heterosexual women using FTC/TDF to prevent acquisition of HIV-1. The risk of HIV-1 acquisition is increased two-fold in women during pregnancy and the early post-partum period[8], and thus PrEP could be an important strategy to reduce risk of HIV-1, if demonstrated to be safe. Accordingly, it is important to assess whether FTC/TDF when used as PrEP by lactating mothers is excreted into breast milk and absorbed by the nursing infant in biologically significant concentrations. Determining the concentration of FTC/TDF infants are exposed to and subsequently absorbed via breast milk when taken daily as PrEP by lactating women is needed to evaluate the benefits and risks of maternal FTC/TDF PrEP use during lactation. Specially, the concentration of FTC and TDF excreted into and absorbed by nursing infants via breast milk of lactating women using FTC/TDF PrEP for HIV-1 prevention has not been studied. This protocol describes an Interventional, open-label, single-arm, repeat-dose study among heterosexual HIV-1 uninfected lactating women to determine the magnitude and extent of infant exposure to daily FTC/TDF via maternal breast milk when taken over one week as PrEP by lactating women.

Design: Interventional, open-label, single-arm, repeat-dose, pharmacokinetic study
Up to 10 days of follow-up per mother-infant pairs

Population: HIV-1 uninfected lactating mother-infant pairs

Study Site: Thika, Kenya and Kampala, Uganda Partners PrEP Study sites

Approach: We will enroll up to 50 mother-infant pairs to characterize infant drug absorption for infant ages 0-24 weeks postpartum. We will conduct quantitative analyses to describe the magnitude and extent of infant exposure to FTC/TDF PrEP through breast milk when used by lactating women. The study approach is detailed in Figure 1.

Figure 1. STUDY APPROACH

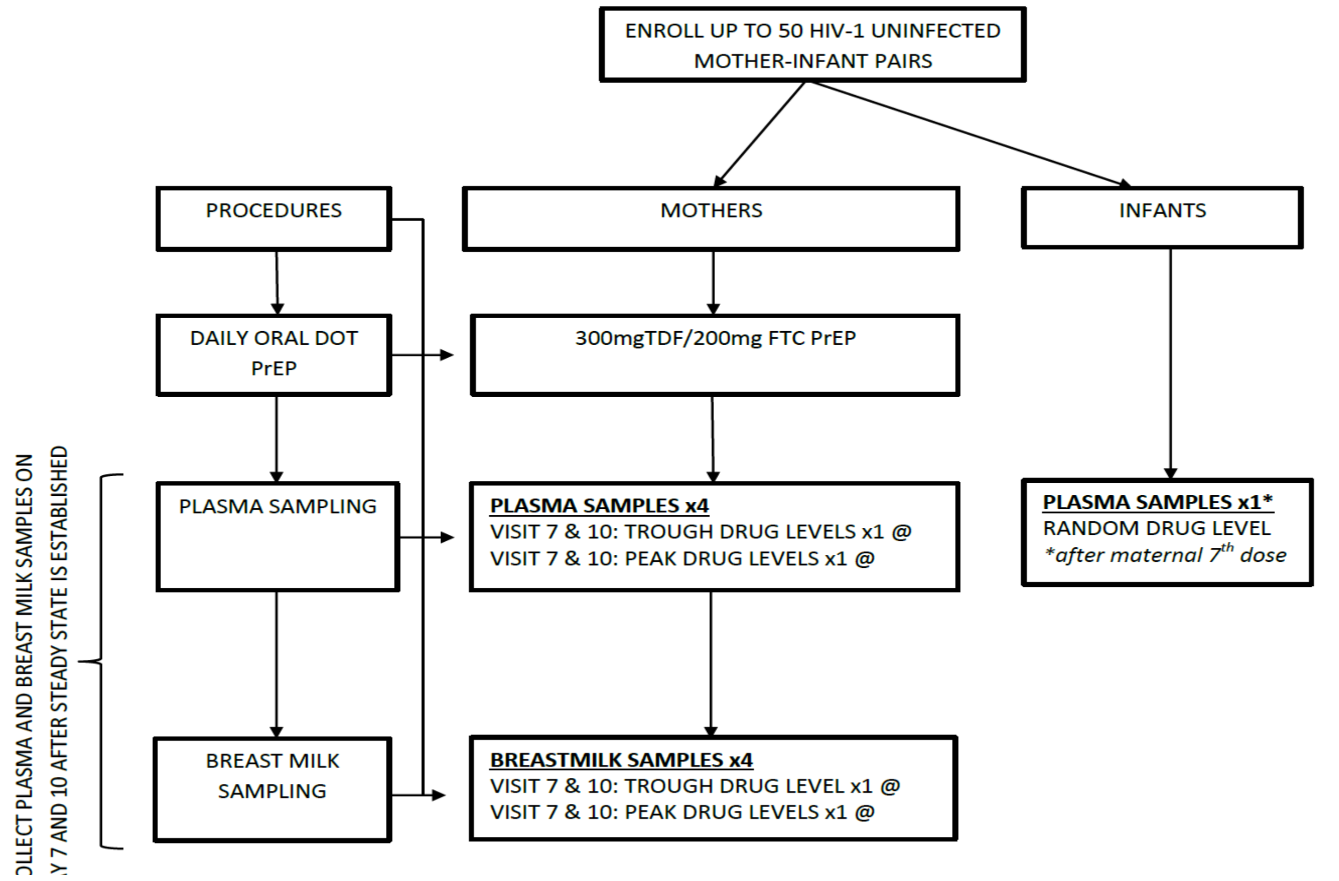

Specific Aim:

To quantify the steady state concentrations of tenofovir (TFV) and emtricitabine (FTC) in infant plasma after FTC/TDF PrEP exposure through maternal breast milk.

Decisions about the safety of breast feeding during maternal ingestion of drugs require knowledge of the amount of drug which might be present in breast milk. Understanding the concentration of FTC/TDF infants are exposed to and subsequently absorbed via breast milk when taken as PrEP by lactating women is needed to permit a thorough evaluation of the benefits and risks of maternal FTC/TDF PrEP use during lactation. We will measure infant plasma, maternal plasma, and breast milk FTC and TFV concentrations in an open-label, repeat dose study of daily FTC/TDF PrEP among HIV-1-uninfected lactating mother-infant pairs.

The primary outcomes of this study will be the steady state infant plasma FTC and TFV levels, infant plasma to maternal breast milk FTC and TFV concentration ratios, and maternal breast-milk to plasma FTC and TFV concentration ratio. A single infant plasma sample will be collected after the maternal 7th PrEP dose. Four maternal plasma and breast-milk samples each will be collected, for trough and peak levels immediately before administration of the 7th and 10th dose and 1-2 hours after the 7th and 10th dose, respectively, two samples at each visit.

II. Background and Rationale.

More than 2.5 million persons are infected with HIV-1 annually, the majority in sub-Saharan Africa, the region with the highest prevalence.[9] In sub-Saharan Africa, the majority of new HIV-1 infections occur in women of childbearing age.[9] Pregnancy and postpartum breastfeeding represent periods of heightened HIV-1 risk for women and for their infant if acute HIV-1 infection occurs.[10] HIV-1 uninfected women who desire pregnancy with a partner who is HIV-1 infected or at high risk of being infected require protective strategies for themselves and their future children. Novel, effective HIV-1 prevention strategies remain urgently needed, particularly those that are deliverable to and useable by high-risk populations. Daily oral pre-exposure prophylaxis (PrEP) for uninfected persons to prevent HIV-1 acquisition[1, 3, 4], peri-coitally dosed tenofovir gel[11], and antiretroviral treatment (ART) to reduce the infectiousness of HIV-1 infected persons[12] are the most promising new approaches for decreasing HIV-1 spread. These antiretroviral-based strategies provide an HIV-1 prevention option for women who desire conception or pregnancy and that is under their control; other available HIV-1 prevention strategies of condoms and abstinence, do not allow for conception.

PrEP protects against heterosexual transmission of HIV-1

Pre-exposure prophylaxis (PrEP) with tenofovir disoproxil fumarate (TDF) alone or in combination with emtricitabine (FTC/TDF) reduces the risk of HIV acquisition in high-risk HIV-uninfected adults in a diverse geographical and at risk populations. Clinical trials have demonstrated protective efficacies of 44-75% in randomized comparisons and ~90% in case-control analyses of participants with high PrEP adherence.[1-4] In the Partners PrEP Study, a phase III, placebo-controlled trial of oral TDF and combination FTC/TDF PrEP among HIV-1 uninfected partners in 4747 HIV-1 serodiscordant in Kenya and Uganda, compared to placebo, FTC/TDF and TDF reduced risk HIV-1 acquisition in both men and women by 75% and 67%, respectively (Table 1). The rate of serious medical events was similar across the study arms. Further evidence for the efficacy of PrEP in the Partners PrEP Study is provided by a case-cohort analysis. For subjects on the active PrEP arms who acquired HIV-1 after randomization, 31% had tenofovir detected in a plasma sample at the seroconversion visit compared with 82% of a randomly-selected samples from a subset of subjects who did not acquire HIV-1, verifying overall high adherence in the trial and demonstrating that seroconverters had low adherence (Table 2). Importantly, having detectable tenofovir in plasma was associated with a relative risk reduction for acquiring HIV-1 of 86% (TDF, p<0.001) and 90% (FTC/TDF, p=0.002) – further emphasizing high protection against HIV-1 for those who were PrEP-adherent

Table 1. Partners PrEP Study efficacy results – July 2011

	TDF	FTC/TDF	Placebo
**HIV-1 incidence,** per 100 person-years	0.65	0.50	1.99
**HIV-1 protection efficacy, vs placebo**	**67%**	**75%**	
95% CI	(44-81%)	(55-87%)	
p-value	<0.001	<0.001	

Table 2. Case-cohort analysis

	**Number / total samples (%) with tenofovir detected**	
	**Case at seroconversion**	**Cohort**
**TDF arm**	6 / 17 (35.3%)	363 / 437 (83.1%)
**FTC/TDF arm**	3 / 12 (25.0%)	375 / 465 (80.6%)

The Partners PrEP Study results reinforce findings of other recent PrEP studies: in CAPRISA 004 peri-coital tenofovir vaginal gel reduced HIV-1 risk by 39% (95% CI 6-60%, p=0.017) among 889 South African women[11]; in iPrEx, daily oral FTC/TDF reduced HIV-1 risk by 44% (95% CI 15-63%, p=0.005) among 2499 men who have sex with men,[3] and oral FTC/TDF reduced HIV-1 risk by

63% (95% CI 22-83%, p=0.01) among 1200 young heterosexuals from Botswana in the TDF2 study[4]; in Bangkok tenofovir study, daily TDF reduced HIV-1 risk by 49% (95% CI 9·6-72·2; p=0.01) among 2413 HIV-1 uninfected adult injection drug users.[2] Across PrEP studies, adherence has been a key predictor of efficacy; largely explaining the lack of HIV-1 protection found among African women in the FEM-PrEP (using FTC/TDF)[13] and VOICE trials (using TDF, FTC/TDF, and tenofovir gel) in which <30% had detectable tenofovir in plasma.[14]

Pregnancy and postpartum breast feeding are periods of heightened HIV-1 risk and women in reproductive years are a target population for PrEP to prevent HIV-1 acquisition

Pregnancy and postpartum period represent periods of heightened HIV-1 risk for women and for their infant if acute HIV-1 infection occurs. Published observational data from sub-Saharan Africa indicate incidence rates during pregnancy and postpartum period as high as 16.8 and 4.7 per 100 person-years in some settings[8, 15-18], respectively (Table 3). Gray et al using data from a cohort of HIV-1 serodiscordant couples in Rakai, Uganda and Mugo et al using data from HIV-1 uninfected women from 7 countries in east and Southern Africa both estimated a 2-fold higher risk of HIV-1 acquisition during pregnancy than non-pregnancy periods[8, 17]. In a systematic review and meta-analysis of observational data, pooled incidence rates during pregnancy, postpartum period, and pregnancy and/or postpartum periods) were 5.1 (95% CL: 3.4 – 6.7), 2.7 (95% CI: 1.6 – 3.8), and 4.1 (95% CI: 3.1 – 5.1), respectively[19]. Because early HIV infection is associated with increased HIV-1 viraemia[20, 21], incident maternal infections might increase mother-to-child HIV transmission. Peri-conception periods, when condom use is reduced, are periods of high HIV-1 risk and women continue to be at high HIV-1 risk if condom use is not resumed during pregnancy and lactating periods. Antiretroviral-based interventions including PrEP have the potential to impact the HIV-1 risk in these periods of heightened risk in women and their use is likely to continue through postpartum breast feeding.

Table 3. Studies of HIV incidence rates during pregnancy and postpartum

Author	Person-years	Incidence/100 person-years
	**During Pregnancy**	
Gray et al 2005 AIDS	997	2.3 (1.5, 3.5)
Mugo et al 2012 AIDS	231	7.4 (4.3, 11.8)
Kieffer et al 2011 J AIDS	246	16.8 (12.7, 21.7)
Moodley et al, 2009 AIDS	679	10.7 (8.2, 13.1)
Taha et al, 1998 AIDS	337.9	8.0 (5.0, 11.0)
Kinuthia eta , 2010 Curr HIV Res	779.4	6.8 (5.1, 8.8)
	**During Postpartum**	
Gray et al, 2005 AIDS	3043	1.3 (0.9, 1.8)
Mbizvo e t al, 2001 Cent Afr J Med	727.94	4.7 (3.2, 6.5)
Humphrey et al, 2006 AIDS	7762.7	3.5 (3.1, 3.9)
Braunstein et al, 2011 Sex Trans Dis	375	3.2 ((1.4, 5.1)

Tenofovir use during pregnancy and infant outcomes

Clinical trials of PrEP excluded pregnant women and women who became pregnant during the study stopped study drug. In an early drug development study involving infant macaque model of pediatric human immunodeficiency virus (HIV) infection, Van Rompay et al using doses of reverse transcriptase inhibitor 9-[2-(phosphonomethoxy) propyl] adenine (PMPA; tenofovir) ranging from 4 to 30 mg/kg of body weight administered subcutaneously once daily to 39 infant macaques for a short period of time (range, 1 day to 12 weeks), no adverse effects on infant macaques health or growth were observed.[22] Moreover, even chronic administration of a low dose of PMPA (10 mg/kg) starting at birth was not associated with any adverse health effects after 5 years of daily treatment[22]. Data on PrEP use during pregnancy available from HIV-1 uninfected women who used PrEP during peri-conception periods and early in the first trimester of the Partners PrEP Study shows no statistically significant differences in pregnancy and infant outcomes for those exposed to PrEP versus placebo (Table 4), including infant length, weight, and head

circumference over one year of follow-up after birth.[23] In addition, there is safety data on HIV-1 infected women who have used FTC/TDF during pregnancy. In the large DART trial of routine vs. clinically-driven laboratory monitoring for HIV-1 care, Gibb et al. compared birth and growth outcomes between infants with 0% of *in utero* time with tenofovir exposure versus ≥90% of *in utero* time with tenofovir exposure.[24] After following children for at least 12 months (median follow up time=25 months), there were no differences in renal function, weight or height among tenofovir exposed versus unexposed children. Another source of data from a large US Antiretroviral Pregnancy Registry on 1,219 pregnancies with first-trimester exposure to the FTC/TDF have shown no increase in overall birth defects compared with the general population rates[25].

Table 4. Pregnancy incidence and outcomes, Partners PrEP Study

	TOTAL	TDF	FTC/TDF	Placebo
Number of pregnancies	288	112	80	96
Pregnancy incidence, per 100 woman-years	10.3	11.9	8.8	10.0
P-value, vs. placebo		*0.19*	*0.40*	
Pregnancy outcome<br>Live birth<br><br>Pregnancy loss	 167 (64%)<br><br>95 (36%)	 73 (71%)<br><br>30 (29%)	 40 (54%)<br><br>34 (46%)	 54 (64%)<br><br>31 (36%)
P-value, vs. placebo		*0.35*	*0.26*	

Taken in aggregate, these data suggest that short-term PrEP is safe in pregnancy and FTC is similar to 3TC which has safety record for during pregnancy and postpartum breast feeding but the data on tenofovir are largely limited to teratogenicity and most did not directly assess PK and safety (given safety profiles among adults), among infants exposed to tenofovir in utero. Specifically, the data are incomplete for infants of HIV-1 uninfected lactating women using PrEP where tolerance/toxicity and not teratogenicity is the main focus.

Rationale for evaluation of PrEP among breastfeeding women

Pregnancy and postpartum breast feeding represent periods of heightened HIV-1 risk for women and for their infant if acute HIV-1 infection occurs[8, 15-18, 26]. Women at the greatest risk for HIV-1 acquisition are in their child-bearing years, during which they face HIV-1 acquisition risk in settings where fertility is high such as Sub-Saharan Africa. After demonstration of PrEP efficacy and safety in the general population, study of the safety of PrEP use during lactating period is needed to inform guidelines and recommendations for PrEP use by lactating women. The benefits of breastfeeding on infant morbidity and mortality are well documented[27, 28]; closely associated with survival and early cessation of breastfeeding is associated with increased infant mortality and morbidity in many developing countries.[27-31] Antiretroviral-based interventions including PrEP are powerful novel tools that could impact HIV-1 risk during pre-conception, pregnancy and lactating periods[1-4, 11, 12]. However, maternal adherence to PrEP medication may be compromised for fear of exposing their infant to the medication through the breast milk. In CAPRISA 004[11], Mathews et al reported that women with pregnancies (n= 53) were less adherent to study product (1% Tenofovir Vaginal Gel or placebo) with median adherence of 50% compared to women without pregnancies (n =815) with median adherence of 60% (adjusted odds ratio= 0.52, 95%CI 0.41–0.66, p<0.0001)[32]. Current guidelines for PrEP use by pregnant and lactating women are compromised by the paucity of data, as clinical trials of PrEP excluded pregnant and breastfeeding women. Experience with tenofovir pharmacokinetics in pregnancy and postpartum breast feeding outside of clinical trials has largely been among HIV-1 infected women for PMTCT but because a majority of these infants were exposed to circulating drugs via the placenta and in addition to oral TDF,[33] their pharmacokinetic parameters likely represent a combination of both vertical- and oral-administration pharmacokinetic patterns.

Infant exposure to emtricitabine and tenofovir disoproxil fumarate via breast milk lactating women using FTC/TDF PrEP

Like other nucleotide analog reverse transcriptase inhibitors (lamivudine and zidovudine),[34, 35] both emtricitabine and tenofovir, have been shown in small studies to be excreted into breast milk. In a previous study of the transfer of tenofovir in breast milk of two rhesus macaques, tenofovir was detected in the milk of both animals, but the peak concentrations (~0.6 to 0.8 μg/ml) were only ~2 to 4% of those detected in serum.[36] In the ANRS 12109 TEmAA (Tenofovir/Emtricitabine in Africa and Asia) study among five Ivorian mothers who chose to exclusively breast feed their infant and were administered one tablet of nevirapine (200 mg) plus two tablets of TDF (300mg)-FTC (200 mg) at the start of labor and one FTC/TDF daily tablet for 7 days postpartum, median tenofovir and emtricitabine breast milk doses represented 0.03% and 2%, respectively, of the proposed oral infant doses.[37] In that study, plasma neonatal tenofovir/emtricitabine levels were estimated by simulation from predicted tenofovir and emtricitabine breast milk doses and were not directly measured. However, it is difficult to predict drug transfer into milk based only on physicochemical properties and current models are limited in prediction of milk concentrations of drugs in humans.[38]

Summary of rationale for the study

The standard for use of drugs during pregnancy and lactation is very high due to safety concerns for unborn or breasting infant. The primary goal of this study is to quantify the magnitude of FTC/TDF that breast feeding infants are exposed to when it is used as pre-exposure prophylaxis by their HIV-1 uninfected mothers. The primary measure will be infant plasma drug concentration. Women are at the greatest risk for HIV-1 acquisitions are in their child-bearing years, during which they face HIV-1 acquisition risk. A serious consequence of HIV-1 acquisition during pregnancy, delivery, and postpartum breastfeeding is vertical transmission of HIV-1 to their child because of high viraemia associated with acute HIV-1 infection. Therefore women are natural target population for pre-exposure prophylaxis to reduce HIV-1 risk during periods of heightened risk including lactation. Inadvertently, nursing infants for HIV-1 uninfected women taking PrEP for HIV-1 infection will be indirectly exposed to FTC/TDFvia breast milk. Transmission of biologically significant drug concentrations to nursing infants through breast milk could occur. A common reason for the cessation of breastfeeding is the use of medication by the nursing mother and advice by her physician to stop nursing. This study is intended to supply the pediatrician, obstetrician, and family physician with data concerning the excretion of FTC/TDF into human milk and absorbed by the nursing baby. This information is important not only to protect nursing infants from untoward effects of maternal FTC/TDF medication but also to allow effective pharmacologic chemoprophylaxis of breastfeeding mothers to reduce their HIV-1 risk. The benefits of breastfeeding are well known and undisputed, so clinician so clinicians should counsel mothers on both the clinical benefits and potential adverse effects to them and their infants before any recommendation for weaning. Thus, data gained from this study will permit a thorough evaluation of the benefits and risks of maternal FTC/TDF PrEP use during lactation.

Although current aggregated data on FTC/TDF safety show the frequency of adverse effects among infants exposed to FTC/TDF during pregnancy are similar to the general population rates, these data are incomplete for HIV-1 uninfected women using FTC/TDF PrEP to prevent acquisition of HIV-1 and are largely limited to fetal teratogenicity and not toxicity. Specifically, there are no published data from infants who have been breastfed while the HIV-1 uninfected mothers have taken FTC/TDF PrEP. The proposed study will provide the first empirical data on the magnitude and extent of infant absorption of FTC/TDF via breast milk when taken as PrEP by lactating women.

III. STUDY METHODS

Overall Design

This is an interventional, open-label, single-arm, short-duration, repeat-dose pharmacokinetic study of daily FTC/TDF PrEP among HIV-1-uninfected lactating mother-infant pairs. Women and infants will be exposed to 10 days of FTC/TDF PrEP – sufficient to reach steady-state but discontinuing thereafter. The overall goal is to quantify the magnitude and degree to which breastfeeding infants are exposed to FTC/TDF when used as PrEP by HIV-1-uninfected lactating women. We will conduct quantitative measurements and analyses of infant plasma drug concentrations, infant-plasma to breast-milk and breast-milk to maternal-plasma drug concentration ratios to characterize FTC and TDF transmission to breast feeding infants.

Aim **To quantify the steady state concentrations of tenofovir (TFV) and emtricitabine (FTC) in infant plasma after FTC/TDF PrEP exposure through maternal breast milk.**

Decisions about the safety of breast feeding during maternal ingestion of drugs require knowledge of the amount of drug which might be present in the milk. Understanding the concentration of FTC/TDF infants are exposed to and subsequently absorbed via breast milk when taken as PrEP by lactating women is needed to permit a thorough evaluation of the benefits and risks of maternal FTC/TDF PrEP use during lactation. We will measure infant plasma, maternal plasma, and breast milk FTC and TFV concentrations in an open-label, repeat dose study of short-term daily FTC/TDF PrEP among HIV-1-uninfected lactating mother-infant pairs.

<u>Hypothesis</u>: *Steady state TFV and FTC concentrations will be substantially low in infant plasma after PrEP exposure through maternal breast milk.*

The primary outcomes of this aim will be the steady state infant plasma FTC and TFV levels, infant plasma to maternal breast-milk FTC and TFV concentration ratio, and maternal breast-milk to plasma FTC and TFV concentration ratio. A single infant plasma sample will be collected after the maternal 7th PrEP dose. Maternal plasma and breast-milk samples for trough and peak drug levels will be sampled immediately before administration of the 7th and 10th doses and 1-2 hours after the 7th and 10th doses, respectively.

Population

HIV-1-uninfected mother-infant pairs will be recruited from the Partners PrEP Study sites in Kampala, Uganda and Thika, Kenya. The sample size of up to 50 pairs will be achieved across the two study sites, with numbers per site to be determined based on rate of recruitment and other study performance metrics. We anticipate that 25-30 mother-infant pairs (i.e., approximately half, for this two-site study) will be recruited at each site.

Eligibility

For infant's mother and father

- Able and willing to provide informed consent for the infant to participate in the study
- Of legal age ≥18 years to consent

For HIV-1 uninfected mother, in addition to the criteria noted immediately above:

- Willing to provide breast milk samples and breastfeed during the duration of the study 0-24 weeks postpartum
- Breastfeeding an infant
- HIV-1 uninfected based on negative HIV-1 rapid tests, both at study screening and at the enrollment visit
- Adequate renal function, defined by normal creatinine levels and estimated creatinine clearance ≥60 mL/min
- Not infected with hepatitis B virus, as determined by a negative hepatitis B surface antigen test
- Not currently using PrEP
- Enrollment of individuals with active and serious infections or active clinically significant medical problems will be at the discretion of the site investigator
- *Note: single mothers are eligible to participate in this study. Where possible the father's permission will be obtained. When the father is unknown, incompetent, deceased, or not reasonably available, or when only the mother has the legal responsibility for the care and custody of the child, the infant participant will be based on the mother's consent and documentation will be added to file*

For infant

- Infant born to eligible women
- Age 0-24 weeks
- Otherwise infant has no serious infections or active clinically significant medical problems

Exclusion criteria

- Women breastfeeding more than one child
- Preterm babies or infants with low birth weight (i.e. ≤2000mg)

Sample size

Up to 50 mother-infant pairs are expected to participate in this open-label, repeat-dose study. Sampling will be stratified on infant age: 0 to <12 weeks and 12 to 24 weeks, with up to 25 mother-infant pairs per age stratum. The proposed sample size is sufficient to permit a thorough evaluation of infant drug exposure while capturing changes in breast milk composition over varying postpartum weeks as well as changes in infant feeding, absorption, and clearance patterns -factors which contribute to the time- and phase-dependent variation of drug excretion into milk and subsequent infant absorption. The proposed sample size is consistent with samples sizes used in similar antiretroviral pharmacokinetic studies.[34]

Ethical requirements for studies involving lactating women and infants

In the proposed study, the likelihood of serious risks are expected to be minimal for this 10 day study and the study is likely to yield generalizable knowledge beyond the study period. The Postpartum breastfeeding is a time of heightened HIV-1 acquisition risk and women infected during postpartum breastfeeding have increased risk of transmitting HIV-1 to their infant during acute infection[8, 15-18, 26]. FTC/TDF was FDA-approved for use by HIV-1 infected individuals in 2004 following standard protocols for safety testing and in 2012 for use by HIV-1 uninfected individuals[5]. Increasingly, TDF and FTC are becoming an integral part of the antiretroviral regimens used in

pregnant and post-partum women in many countries. Evidence from randomized clinical trials of HIV-1 uninfected women with first trimester exposure to TDF and FTC show no risk of birth defects or poor growth outcomes.[23] In addition, data from a large US Antiretroviral Pregnancy Registry on 1,219 pregnancies with first-trimester exposure to the FTC/TDF have shown no increase in overall birth defects compared with the general population rates[25]. Two studies have attempted to assess drug levels in infants specifically from breast milk exposure from their HIV-1 infected mothers. First, the ANRS 12109 TEmA study[37] enrolled five Ivorian HIV-1 infected women who chose to exclusively breast-feed their infants and were administered one tablet of Nevirapine (200 mg) plus two tablets of TDF (300 mg)-FTC (200 mg) at the start of labor and one FTC/TDF FTC/TDFdaily tablet for 7 days postpartum. Median TFV and FTC breast milk doses represented 0.03% and 2%, respectively, of the proposed oral infant doses. However, infant plasma drug levels were only estimated by simulation and not directly measured. Some of the model assumptions used in that study may be unrealistic and FTC/TDF was not at steady state. Second, the HPTN 057 protocol[39], a multicenter study of the international maternal pediatric adolescent AIDS Clinical Trials Group (IMPAACT), administered 600 mg doses TDF in labor with no infant dosing to HIV-1 infected women. The goal was to assess fetal and newborn TDF exposure from labor through the end of the first week of life to protect against intrapartum and early postpartum/breast feeding HIV transmission. However, because the infants were exposed both transplacentally and through breast milk, the observed drug levels are likely a combination of pharmacokinetics of both routes. Importantly, the study only used TDF as single dose and not FTC/TDF. Our study is different from HPTN 057 protocol, in that we will use FTC/TDF and drug levels will be assessed after achieving steady state in the mothers at which drug absorption and elimination will be at equilibrium. Thus, this study will offer complimentary and new information. Additional studies have also assessed the FTC/TDF drug excretion in context of PTMC including Flynn et al[33] that evaluated the pharmacokinetics and safety of single-dose FTC/TDF in HIV-1-Infected pregnant women and their Infants. However, the focus in these trials were transplacentally transferred drugs and not through breast milk. Additional limitation for extrapolation of these data is that there is differential clearance of plasma TFV/FTC between that transferred across the placenta and doses received orally through breast milk because of the way gastric pH affect drug bioavailability. There has been extensive use and study of TDF (and FTC) in pregnant and breastfeeding HIV-1 infected women, including the recently-published results from the DART trial [40]. Thus, data from HIV-1 infected women provide important background for the proposed study, more than sufficient to permit the proposed work to be done safely. However, data from HIV-1 infected women, who may have different metabolism or excretion of medication, given disease and concurrent antiretroviral medications, mandate assessment of this important issue in HIV-1 uninfected women and their infants. The involvement of these vulnerable populations is critical to answer the research questions posed. Any benefits derived from this study are likely to have most impact on the population from which these women and infants are drawn. Research among pregnant or lactating women and children requires special ethical considerations.

For research to move forward in logical, stepwise increments, a framework has recently been described to identify specific steps needed to move clinical research into populations of pregnant and lactating women (Table 5).[41]

For FTC/TDF PrEP, the next incremental step for safety assessment to inform the risks and benefits of PrEP use in lactating women is a time-limited evaluation of medication excretion in breast milk and infant absorption. Given the high risk of HIV-1 acquisition during pregnancy and postpartum breastfeeding and risk of transmission to the nursing infant in the event of acute HIV-1 seroconversion as well as the difficulties of condom use negotiation for women, the high efficacy of FTC/TDF in reducing HIV-1 acquisition combined with an excellent safety profile,

Table 5. Recommended considerations prior to the evaluation of investigational drugs in pregnancy and rationale for addressing each consideration (adapted from *Beigi et al. 2013*)

Question	Response for the case of FTC/TDF safety testing during lactation
1. What is the rationale for evaluating the drug in lactation?	Pregnancy and lactation is a time of high risk for HIV-1 infection and possible vertical HIV-1 transmission in the event of maternal acute HIV-1 infection.
2. Has testing for animal fertility, reproductive, and developmental toxicity been conducted?	Yes. FTC/TDF was FDA-approved for use by HIV-1 infected individuals in 2004 following standard protocols for safety testing. The approval for use by HIV-1 uninfected individuals was in 2012.
3. Have signals been identified in *in vitro* or animal testing	No signals of safety concerns have been identified in *in vitro*, animal or human observational data
4. Have any drugs of the same class been evaluated in preclinical or clinical testing during pregnancy?	Most NNRTIs are approved for use by HIV-1 infected women, including use during pregnancy and lactation; only TDF/FTC is approved for use by HIV-1 uninfected women to protect against HIV-1 infection
5. Are there known of potential risks to the mother or fetus?	Data from inadvertent exposure during first trimester do not demonstrate any risks
6. Are there known potential benefits to the mother or fetus?	Yes. The mother and infant will benefit from protection against HIV-1 infection
7. Can a case be made for the prospect of direct benefit to the mother or fetus?	Yes. Postpartum lactation is a time of heightened HIV-1 acquisition risk. Women infected during postpartum breastfeeding have increased risk of transmitting HIV-1 to their infant during acute infection.
8. Do alternatives exist to use of this drug in pregnancy?	No. This is the only drug currently approved for use among HIV-1 uninfected women for HIV-1 prevention
9. Which trimester(s) would be exposed with use	Infants will be exposed during breast feeding. No trimester will be exposed as pregnant women will not participate.
10. How is the drug eliminated?	The drug is eliminated through the kidney and creatinine clearance is recommended to be calculated prior to initiating therapy.
11. Are clinical data available on pregnancy outcomes following exposure, and what is the quality of this evidence?	a) High quality evidence from randomized clinical trials of HIV-1 uninfected women with inadvertent first trimester exposure to TDF shows no risk of birth defects of poor growth outcomes.<br>b) Evidence from observational cohort studies among HIV-1 infected women using tenofovir for HIV-1 treatment have shown no clinically significant growth delays in infants exposed to tenofovir in utero.

indicates that FTC/TDF PrEP is a promising intervention for HIV-1 prevention among lactating women, and warrants evaluation of breast milk FTC and TDF concentrations and absorption among infants.

Recruitment

Accrual will continue until up to 50 evaluable mother/infant are recruited into the study overall. Recruitment will be over in 6 months. A fully evaluable mother-infant pair is defined as one for which a complete set of specimen is obtained as specified in procedures and table 6. Mother-infant pairs that are not fully evaluable are technically not 'replaced' in the study, as all participants exposed to the study drug will remain in the study for safety monitoring and complete follow-up as originally scheduled, even if dosing is discontinued early; however these mother infant pairs will not count toward the targets sample size. The Thika, Kenya and Kampala, Uganda study sites have established local recruitment and screening methods that ensure efficiency in identifying research participants for the local study setting and target study population, including for research involving infants. The Thika, Kenya and Kampala, Uganda sites staff are highly trained to recruit from communities their research clinic are based in and are experienced in both individual and couples counseling. Recruitment strategies will include partnering with antenatal, postnatal, and immunization clinics, and other community-based organizations for referral of potential participants. Mother-infant pairs may also be recruited for possible inclusion in the study from other activities conducted at the Thika, Kenya and Kampala, Uganda study sites. Trained study staff will approach potential participants that may have been identified at the collaborating organizations.

All efforts will be made to maintain potential participant's privacy and confidentiality and to minimize potential coercion or the appearance of coercion. Contact persons at the collaborating organizations will be trained on aims of study and in handling of potential study participants. All screening procedures will be conducted at the study clinic. Trained study staff will inform the potential participants that their participation is completely voluntary, and that they will continue accessing care at their regular health facility if they choose not to participate in this study. Approval for all the recruitment procedures and materials will be sought from all relevant IRBS.

The screening process will proceed in a step-wise manner until either all screening procedures are completed or the mother-infant pair is determined to be ineligible. There is no time limit on the screening process.

For the mother-infant pairs found to be eligible for the study, informed consent for study participation and enrollment in the study may proceed on the same day when eligibility is determined.

Study procedures

Specific study procedures are detailed in Table 6. Visits will take place at screening and enrollment, and then daily thereafter, for up to 10 days.

Procedure for HIV-1 uninfected women

At screening, demographic, and breastfeeding information will be collected along with laboratory results to establish participant eligibility (serum creatinine, hepatitis B surface antigen, and HIV-1 rapid testing according to national algorithms).

At enrollment, HIV-1 testing will be performed for women to confirm eligibility (HIV-1 seronegative at the time of study start). HIV-1 uninfected women who have symptoms potentially consistent with acute HIV-1 infection (fever, rash, pharyngitis) will have enrollment deferred for 2 weeks at which time repeat serologic testing will be performed (and, if positive, will result in study exclusion). HIV-1 uninfected women will have a pregnancy test to confirm eligibility (must be not pregnant to complete enrollment).

At enrollment and then each daily follow-up visit, study mothers will be prescribed oral directly observed therapy (DOT) FTC/TDF PrEP. Participants will be counseled (for compliance /adherence to study procedure, drug toxicity, risk reduction) and complete breast feeding and medical history interviews with staff counselors.

During daily follow-up, HIV-1 uninfected women will complete a short quantitative interview to capture breasting pattern of the infants and proportion of infant feeding due to breast milk, adverse events, and concomitant medication use. Maternal blood and breast milk samples for drug assays will be collected at the 7th and 10th FTC/TDF dose (details in sample collection section below).

All data collections will be conducted in a private room in the participant's preferred language, according to the participant's fluency and preference. Development of data collection tools has been guided by our experience collecting clinical data for the Partners PrEP Study at multiple sites in Kenya and Uganda.

Participant retention and withdrawal

For this pharmacokinetic study, retention efforts will strive to achieve maximum compliance to study visit schedules to ensure the highest possible adherence to study medication. The Thika, Kenya and Kampala, Uganda sites are highly experienced in research participant follow-up and have developed retention methods tailored to and most efficient for their local study settings. Retention activities will include explanation of the study visit schedules and procedural requirements during the informed consent process and re-emphasis at each study visit, collection and updating of locator information, and use of appropriate and timely visit reminder mechanisms (including phone calls and text messages), and home visits for participant. To provide complete information at the end of the study, efforts will be made to have a final follow-up visit for each participant.

Participants may voluntarily withdraw from the study for any reason at any time. The site Investigator also may withdraw participants from the study in order to protect their safety and/or if they are unwilling or unable to comply with required study procedures.

HIV-1 testing

HIV-1 antibody testing – for women at screening and enrollment, will be performed in line with national HIV-1 testing algorithms for Kenya and Uganda. At enrollment, prior to maternal FTC/TDF dosing, the Infant will have an antibody test using parallel rapid HIV-1 tests in accordance with the national algorithms Kenya and Uganda. For mothers found to be infected with HIV-1 at screening, their infants will have a PCR to test for HIV-1 infection.

Sample collection

The frequency and volume of blood draws are defined in Table 6. Blood and breast milk specimens will be collected as per the study procedures manual.

Table 6. Procedures for HIV-1-uninfected mother-infant pairs

	S	E/D1	D2	D3	D4	D5	D6	D7	D8	D9	D10
**ADMINISTRATIVE AND REGULATORY PROCEDURES**											
Obtain informed consent	X	X									
Apply inclusion/exclusion criteria	X	X									
Collect/update locator information	X	X	X	X	X	X	X	X	X	X	X
Collect demographic information	X										
**COUNSELING**											
Provide HIV-1 pre and post-test counseling	X	X									
Adherence/compliance counseling		X	X	X	X	X	X	X	X	X	X
Drug toxicity assessment and concomitant medication counseling		X	X	X	X	X	X	X	X	X	X
**CLINICAL PROCEDURES**											
Provide HIV-1 test results	X	X									
Acute HIV-1 assessment		X									
Medical history / symptoms information	X	X	[X]	[X]	[X]	[X]	[X]	[X]			
Perform clinical assessment		X	[X]	[X]	[X]	[X]	[X]	[X]			
Maternal blood specimen [max. 21 mL] (screening laboratory eligibility tests, enrollment sample for HIV-1 test)	X	[X]ǂ									
DOT FTC/TDF PrEP		X	X	X	X	X	X	X	X	X	X
Maternal plasma specimen [max. 10 mL each sampling visit] (4 samples (total) to archive to measure trough & peak FTC & TFV concentrations)								X			X
Whole breast milk specimen [max. 10 mL each sampling visit] (4 samples (total) to archive to measure trough & peak FTC & TFV concentrations)								X			X
Infant plasma specimen [max. 2 mL] (1 sample to archive to measure FTC & TFV concentrations)								X	[X]	[X]	[X]
**BREAST FEEDING DATA COLLECTION**											
Collect breastfeeding information	X	X	X	X	X	X	X	X	X	X	X
**LOCAL LABORATORY PROCEDURES**											
Creatinine and creatinine clearance	X										X
HIV-1 serology (rapid tests)<br>Infant HIV antibody test at enrollment only.	X	X									
HBV surface antigen	X										
Urine pregnancy test		X									

[ ] as indicated; S-refers to screening; E-refers enrollment; D-refers to day

After continuous daily oral DOT FTC/TDF PrEP to achieve drug steady state, a total of four samples each for maternal plasma [maximum 10 mL at each sampling visit] and whole breast milk [maximum approximately 10 mL at each sampling visit] will be collected for steady state trough and peak drug concentrations at study visit 7 and 10. Maternal plasma and breast milk samples for trough FTC and TFV concentration measurements will be concurrently collected (within 30 minutes) immediately before administration of the respective 7th and 10th doses. Samples for peak maternal plasma and breast-milk FTC and TFV concentrations will be concurrently (within 30 minutes) collected within 1-2 hours after the respective 7th and 10th doses,

respectively. In total, four blood samples and breast milk samples each will be collected, two of respective sample at study visit 7 and study visit 10.

Procedures for the infant

The infant will be required to attend at least two study visits including the enrollment visit and the mother's study visit 7. However, mothers will be encouraged to come with their babies at their scheduled visit or if they feel the infant is unwell.

At the enrollment visit, we will obtain a small blood sample by a needle prick on the infant's finger or heel for an HIV-1 antibody test. The study staff will talk with the mother about the HIV-1 test and what it may mean to know the infant's HIV-1 status. Although it is very unlikely for the infant to be HIV-1 infected if their mother is HIV-1 uninfected, on very rare occasions it may be possible for the infant to have HIV-1 exposure from procedures involving medical injections or blood transfusion, or breastfeeding from a woman other than the mother. For infants found to be antibody positive for HIV-1, study staff will counsel the mother. The infant will be referred to the appropriate health facility for further check-up and no further study procedures will be performed on the mother and the infant.

At the mother's study visit 7, one blood sample [maximum 2 mL] will be collected from the infant to measure the infant plasma drug concentrations ingested via maternal breast milk. The blood sample shall be collected within 2 hours of study mother's 7th study drug dose (after maternal drug steady state). At each of the infant's scheduled study visits study staff will perform a clinical assessment. Infant age, gender, and weight will be recoded. If for any reason, infant blood sample cannot be collected on study visit 7, the sample can collected at any visit between visit 7 and visit 10 inclusive but before the end of the study.

Laboratory Procedures
Table 6 shows details of all planned laboratory evaluations, including the relevant objectives, sample, responsible laboratory and the time of sample collection mother-infant pairs.

Procedures for specimen collection, transport, processing, storage and shipping will be included in the study procedures manual. All specimens should be transported to the laboratory within 4 hours of collection. All specimens will be labeled with a unique laboratory accession number which can be linked to the study unique coded identification number. Plasma and breast milk samples will be processed and stored at -70°C and subsequently analyzed by Johns Hopkins Clinical Pharmacology Laboratory, USA. Tenofovir and emtricitabine concentrations will be measured by liquid chromatography-tandem mass spectrometry (LC-MS/MS) previously validated for human plasma and breast-milk.[42-44] The analytic laboratory conforms to international standards of good laboratory practices (GLP) and GLP-like analyses for clinical samples.

Training and Quality Assurance

Demographic, breast feeding, and clinical data will be entered onto standard case report forms. Study staff involved in obtaining informed consent and client interviews, clinical examinations and specimen collection will receive relevant training prior to the commencement of this study. Pre and post training assessments will be conducted. Senior study staff members will continue to review clinical technique and assessment over the study period.

Participant retention and withdrawal

For this pharmacokinetic study, the goal is to achieve high FTC/TDF adherence to reflect maximum possible exposure from standard FTC/TDF PrEP doses. The project will recruit until up to 50 evaluable mother-infant pairs successfully complete all study procedures.

Participants may voluntarily withdraw from the study for any reason at any time. The site Investigator also may withdraw participants from the study in order to protect their safety and/or if they are unwilling or unable to comply with required study procedures. Reasons for withdrawal will be recorded.

PrEP medication

This study will provide open-label PrEP to HIV-1 uninfected lactating women. Only eligible HIV-1 uninfected women will receive study drug in this study. Tenofovir disoproxil fumarate and emtricitabine are reverse transcriptase inhibitors that have been approved for the treatment of HIV-1 infection in humans in Kenya, Uganda, and the United States. A fixed-dose, oral co-formulation of FTC/TDF will be used in this study. The dose of FTC/TDF is the standard dose approved by the U.S. Food and Drug Administration. PrEP will be prescribed for once-daily oral 200m FTC/300mg TDF by directly observed therapy (DOT). Counseling on the medications being used, their side effect profiles, contraindicated concomitant medication, what to do if side effects are experienced will be done. The pharmacy and storage facility will have locked, climate-controlled environments, with controlled temperature to remain within limits allowed by the manufacturer for drug storage. Study medication will be donated by Gilead Sciences.

Adherence

High adherence is critical for PrEP effectiveness in preventing HIV-1 acquisition and understanding the magnitude infant drug exposure for tolerance/toxicity assessment. Daily oral 200mg FTC/300mg TDF PrEP will be administered by directly observed therapy (DOT) to study women at the study site. Brief adherence and compliance counseling will be provided at each scheduled visit.

Discontinuation of PrEP

Use of PrEP may be interrupted by the site Investigator due to safety concerns for the participants (mother and infant), use of concomitant medications that could interfere with PrEP or present a safety concern, or if the participant is unable or unwilling to comply with study procedures. All treatment interruptions will be documented.

Concomitant medication use will be recorded. PrEP will be avoided, per investigator discretion, in individuals receiving ongoing therapy, interferon (alpha, beta, or gamma) or interleukin (e.g., IL-2) therapy, metformin, systemic aminoglycoside antibiotics, amphotericin B, cidofovir, systemic chemotherapeutic agents, other agents with significant nephrotoxic potential, other agents that may inhibit or compete for elimination via active renal tubular secretion (e.g., probenecid).

IV. SAFETY

The Partners PrEP Study demonstrated that PrEP (including FTC/TDF) was safe for use in heterosexual men and women from Kenya and Uganda. There were no statistically significant differences in the frequency of deaths, serious adverse events, adverse events overall or key

laboratory adverse events (specifically, creatinine elevation and phosphorus decrease) for those receiving PrEP compared to those receiving placebo.

All adverse events, for both mother and infant participants will be documented. The severity of clinical symptoms will be scored using the DAIDS Table for Grading the Severity of Adult and Pediatric AEs[45]. Decisions to hold PrEP due to clinical and/or other laboratory safety reasons will be at the discretion of site Investigator. Adverse events will be reported according to relevant IRBs policies and regulations.

Clinical symptoms will be systematically assessed in a structured medical history administered to mother participants. Clinical side effects of TDF and FTC/TDF that have been reported are primarily gastrointestinal, including nausea, vomiting, and flatulence.

V. DATA ANALYSIS

The goal of the analysis is to quantify the magnitude and level of infant exposure to FTC/TDF PrEP through maternal breast milk. Data will be summarized in tabular and graphical forms for presentation. Descriptive statistics will be calculated for demographic and clinical data for mothers and infants. Categorical variables will be summarized as frequencies with proportion, and for continuous measures using mean and standard deviations or medians and ranges, as appropriate. The magnitude of infant exposure to FTC/TDF PrEP will be quantified using infant plasma drug concentrations, infant plasma-to-breast milk and breast milk-to-maternal plasma drug concentration ratios. Antiretroviral concentrations and milk-to-plasma ratios data will be presented as medians and ranges. Additionally, drug exposure index will be computed to estimate the infant daily drug exposure from breast milk feeding.

VI. HUMAN SUBJECTS CONSIDERATIONS

The study protocol, site-specific informed consent forms, participant comprehension questionnaire, education, and recruitment materials, and other requested documents — and any subsequent modifications — will be reviewed and approved by the IRBs/ECs responsible for oversight of research conducted at the study sites. Subsequent to initial review and approval, the responsible IRBs/ECs will review the study at least annually.

Informed consent

Written informed consent will be obtained from each study mother prior to both screening and enrollment. Potential research subject's comprehension of study procedures will be assessed by trained study staff performing the consent process using a comprehension questionnaire. Potential participants don't need to answer all questions correctly to enroll. If in the opinion of the study staff the participant demonstrates poor or inadequate understanding of the study procedures after further explanation and repeat assessment, study procedures may be deferred to another visit and subsequent participation in the study will be at the discretion of the site principal investigator. The fathers' permission will be obtained, where possible. When the father is unknown, incompetent, deceased, or not reasonably available, or when only the mother has the legal responsibility for the care and custody of the child, the mother's consent will be used and documentation will be added to file. Participants will be offered copies of the informed consent forms.

Each study site is responsible for developing study informed consent forms for local use that describe the purpose of screening and of the study, the procedures to be followed, and the risks and benefits of participation, in accordance with all applicable regulations, based on the schedule of procedures in Table 6. Each site also is responsible for translating the forms into local

languages and verifying the accuracy of the translation by performing an independent back-translation. After the local IRBs approvals, consents forms will be returned to UW IRB for final approval and stamping. Study procedures will not begin before the final approvals are obtained from all the relevant IRBs.

Risks

Participants may experience discomfort or pain when undergoing phlebotomy. They also may feel dizzy or faint, and/or develop a bruise, swelling, or infection where the needle is inserted.

Participants may become worried or anxious while waiting for their HIV-1 test results. Participants who learn that they have HIV-1 may experience anxiety or depression related to their test results. At the study sites, HIV-1 counseling will be provided by counselors and clinicians who have been trained in specific issues, including stigma, blame, methods to avoid transmission, and available support services.

Although study sites will make every effort to protect participant privacy and confidentiality, it is possible that participants' involvement in the study could become known to others, and that social harms may result (i.e., because participants could become known as participating in a trial involving medication used for HIV-1 treatment). For example, participants could be treated unfairly or discriminated against, or could have problems being accepted by their families and/or communities.

Risks and side effects related to PrEP include: occurring in a minority of individuals taking PrEP - gastrointestinal intolerance, such as nausea, diarrhea or vomiting, flatulence; rare but serious - lactic acidosis/ severe hepatomegaly with steatosis, renal impairment, including cases of acute renal failure and Fanconi's syndrome (renal tubular injury with severe hypophosphatemia), increase in bone metabolism leading to osteopenia, hypersensitivity reaction. HIV-1 resistance may also emerge in some individuals with undiagnosed primary HIV-1 infection taking only FTC/TDF PrEP because FTC/TDF alone does not constitute a complete regimen for HIV-1 treatment. HIV-1 uninfected subjects who have symptoms potentially consistent with acute HIV-1 infection (fever, skin rash, pharyngitis, fatigue, myalgia, etc) will have enrollment deferred for 2 weeks at which time repeat serologic testing will be performed and, if positive, will result in exclusion from study participation. Serious adverse reactions would be expected to be extremely rare with only 10 days of PrEP use. Risks to infants associated with exposure to FTC/TDF through breastfeeding are unknown but would likely be similar to potential risks in adults.

Benefits

There may be no other direct benefits to participants in this study. However, participants and others also may benefit in the future from information learned from this study. Because of the short duration of this study, a 10-day period of daily PrEP is not expected to offer adequate protection against HIV-1 acquisition to women. On average, at least 2 weeks of daily PrEP would be required to significantly reduce the risk of HIV-1 infection. Women will be counseled to continue using other HIV-1 prevention strategies including condoms and screening and treatment for sexually transmitted infections. All participants will be provided with HIV-1 prevention services.

Care for persons identified as HIV-1 infected at screening

This study may identify persons who are infected with HIV-1 as part of the study screening process. Study staff will provide participants with their HIV-1 test results in the context of post-test counseling. Persons identified as HIV-1 infected during the study screening process, will be referred to local HIV-1 care services and/or other agencies that provide care or access to treatment.

Treatment for injury

Participants will be asked to inform the study staff if they feel they or their infants have been injured because of taking part in the study. Injuries may also be identified during laboratory testing, medical histories, and physical examinations. Treatment for adverse events related to study participation will be provided by the study clinic. If treatment is required that is beyond the capacity of the study clinic, the study doctors will refer the participant to appropriate services or organizations that can provide care for the injury.

Study records

Site Investigators will maintain, and store in a secure manner, complete, accurate and current study records throughout the study. The investigator will retain all study records after completion of the study according to IRB/EC and national regulations. Study records include administrative documentation and regulatory documentation as well as documentation related to each participant screened and/or enrolled in the study, including informed consent forms, locator forms, case report forms, notations of all contacts with the participant, and all other source documents.

Confidentiality

Every effort will be made to protect participant privacy and confidentiality to the extent possible. The study sites will establish a standard operating procedure for confidentiality protection that reflects the local study implementation plan and the input of study staff and community representatives to identify potential confidentiality issues and strategies to address them. In addition to local considerations, the protections described below will be implemented at all sites.

All study-related information will be stored securely at the local study sites. All participant information will be stored in areas with limited access. Data collection, administrative forms, laboratory specimens, and other reports will be identified only by a coded number to maintain participant confidentiality. All records that contain names or other personal identifiers, such as locator forms and informed consent forms, will be stored separately from study records identified by code number. All local databases will be secured with password-protected access systems. Forms, lists, logbooks, appointment books, and any other listings that link participant ID numbers to other identifying information will be stored in a separate, locked file in an area with limited access. The link between the participant's personal identifiers and the code on the participant's data and samples will only be kept at the study sites and only until December 31st, 2015 when the study data collection is expected to be completed. After that time, this link will be destroyed.

Participants' study information will not be released without their written permission, except as necessary for oversight by:

- The study investigators or designees
- Study funders
- Applicable local authorities, e.g., Ministry of Health
- Site IRBs/ECs
- University of Washington
- Any additional study sponsors

Study limitations

While infant feeding practices throughout Uganda and Kenya can be broadly characterized as being universal and predominantly breastfeeding of prolonged duration, factors potentially modified by infant feeding practices of rural versus urban population will need careful interpretation given the small sample size involved. The study population may be also expected to differ on some characteristic from the overall source population due to the exclusion of mothers with multiple live births and the need to live near the study site in order to be able to attend for frequent study visits. In addition, single sample designs can yield imprecise estimates because of random variation; however, this is especially important when the main goal of the study is hypothesis testing for detecting differences between groups. Despite these limitations described, the pharmacokinetic factors to be studied are likely to be robust to cultural and socio-economic differences amongst groups in population studied and those not studied.

Application of study results

This study is intended to provide the pediatrician, obstetrician, family physician and other primary healthcare with data concerning the excretion of FTC/TDF FTC/TDF into human milk and absorbed by the nursing baby. This information is important not only to protect nursing infants from untoward effects of maternal FTC/TDF FTC/TDF medication but also to allow effective pharmacologic chemoprophylaxis of breastfeeding mothers to reduce their HIV-1 risk. Data gained from this study will permit a thorough evaluation of the benefits and risks of maternal FTC/TDF PrEP use during lactation.

Dissemination of study results

Results from this study are not expected to have any direct implications for the subject's health care or for the health care of her baby. Final results that constitute clinically significant or scientifically relevant information will be made available to relevant target audiences. Primary target audiences include study subjects, women of reproductive age in Uganda and Kenya and their health providers. Information will be made available through appropriate local channels such as at academic and public health forums, medical and nursing professional societies, women's community groups and HIV related non-government organizations, ministries of health. Manuscripts will also be submitted for peer review.

Time line

The table below presents the projected study timelines. It is only intended to provide guidance and planning for study execution. The actual milestone dates may vary depending on other factors and study progress.

Commence enrollment	4th Quarter, 2014
Complete enrollment	1st Quarter, 2015
Complete laboratory testing, analysis, and manuscript submission for peer review	December, 2015
Dissemination of findings	2016

Intellectual property rights

The study team jointly will own the intellectual property rights for this study.

References

1. Baeten JM, Donnell D, Ndase P, et al. Antiretroviral Prophylaxis for HIV Prevention in Heterosexual Men and Women. *New England Journal of Medicine* 2012; **367**(5): 399-410.
2. Choopanya K, Martin M, Suntharasamai P, et al. Antiretroviral prophylaxis for HIV infection in injecting drug users in Bangkok, Thailand (the Bangkok Tenofovir Study): a randomised, double-blind, placebo-controlled phase 3 trial. *The Lancet* 2013; **381**(9883): 2083-90.
3. Grant RM, Lama JR, Anderson PL, et al. Preexposure Chemoprophylaxis for HIV Prevention in Men Who Have Sex with Men. *New England Journal of Medicine* 2010; **363**(27): 2587-99.
4. Thigpen MC, Kebaabetswe PM, Paxton LA, et al. Antiretroviral Preexposure Prophylaxis for Heterosexual HIV Transmission in Botswana. *New England Journal of Medicine* 2012; **367**(5): 423-34.
5. FDA approves new combination pill for HIV treatment for some patients. 2012. http://www.fda.gov/NewsEvents/Newsroom/PressAnnouncements/ucm317004.htm (accessed March 9 2013).
6. WHO. Guidance on Pre-exposure Oral Prophylaxis (PrEP) for Serodiscordant Couples, Men and Transgender Women who have Sex with Men at high risk of HIV: Recommendations for use in the context of demonstration projects. 2012. http://apps.who.int/iris/bitstream/10665/75188/1/9789241503884_eng.pdf (accessed March 9 2013).
7. CDC. Interim guidance for clinicians considering the use of preexposure prophylaxis for the prevention of HIV infection in heterosexually active adults. *MMWR Morb Mortal Wkly Rep 2012*; **61**: 586-9.
8. Mugo NR, Heffron R, Donnell D, et al. Increased risk of HIV-1 transmission in pregnancy: a prospective study among African HIV-1-serodiscordant couples. *AIDS* 2011; **25**(15): 1887-95.
9. Schaub S, Wilkins J, Antonovici M, et al. Proteomic-based identification of cleaved urinary beta2-microglobulin as a potential marker for acute tubular injury in renal allografts. *Am J Transplant* 2005; **5**: 729 - 38.
10. SA NDoH. National Antenatal Sentinel HIV and Syphilis Prevalence Survey in South Africa, 2009. Pretoria: National Department of Health. 2010.
11. Abdool Karim Q, Abdool Karim SS, Frohlich JA, et al. Effectiveness and safety of tenofovir gel, an antiretroviral microbicide, for the prevention of HIV infection in women. *Science* 2010; **329**(5996): 1168-74.
12. Cohen MS, Chen YQ, McCauley M, et al. Prevention of HIV-1 Infection with Early Antiretroviral Therapy. *New England Journal of Medicine* 2011; **365**(6): 493-505.
13. Van Damme L, Corneli A, Ahmed K, et al. Preexposure Prophylaxis for HIV Infection among African Women. *New England Journal of Medicine* 2012; **367**(5): 411-22.
14. NIH modifies 'VOICE' HIV prevention study in women: oral tenofovir discontinued in clinical trial. 2011. http://www.nih.gov/news/health/sep2011/niaid-28.htm (accessed Accessed September 28, 2011.
15. Kieffer MP, Nhlabatsi B, Mahdi M, Hoffman HJ, Kudiabor K, Wilfert CM. Improved detection of incident HIV infection and uptake of PMTCT services in labor and delivery in a high HIV prevalence setting. *J Acquir Immune Defic Syndr* 2011; **57**(4): e85-91.
16. Mbizvo MT KJ, Mahomed K, Nathoo K. HIV-1 seroconversion incidence following pregnancy and delivery among women seronegative at recruitment in Harare, Zimbabwe. *Cent Afr J Med* 2001; **47**: 115-8.
17. Gray RH, Li X, Kigozi G, et al. Increased risk of incident HIV during pregnancy in Rakai, Uganda: a prospective study. *Lancet* 2005; **366**(9492): 1182-8.
18. Moodley D, Esterhuizen TM, Pather T, Chetty V, Ngaleka L. High HIV incidence during pregnancy: compelling reason for repeat HIV testing. *AIDS* 2009; **23**(10): 1255-9.

19. Drake AL, Wagner A, Richardson B, John-Stewart G. Incident HIV during Pregnancy and Postpartum and Risk of Mother-to-Child HIV Transmission: A Systematic Review and Meta-Analysis. *PLoS Med* 2014; **11**(2): e1001608.
20. Garcia PM, Kalish LA, Pitt J, et al. Maternal levels of plasma human immunodeficiency virus type 1 RNA and the risk of perinatal transmission. Women and Infants Transmission Study Group. *The New England journal of medicine* 1999; **341**(6): 394-402.
21. Quinn TC, Wawer MJ, Sewankambo N, et al. Viral load and heterosexual transmission of human immunodeficiency virus type 1. Rakai Project Study Group. *The New England journal of medicine* 2000; **342**(13): 921-9.
22. Van Rompay KK, Brignolo LL, Meyer DJ, et al. Biological effects of short-term or prolonged administration of 9-[2-(phosphonomethoxy)propyl]adenine (tenofovir) to newborn and infant rhesus macaques. *Antimicrobial agents and chemotherapy* 2004; **48**(5): 1469-87.
23. Mugo N HT, Celum C, D D, Bukusi E, Were E, et al. Pregnancy incidence and birth outcomes among African women in a clinical trial of pre-exposure prophylaxis: the Partners PrEP Study. Abstract #WEAC0101. In: *7th IAS Conference on HIV Pathogenesis, Treatment and Prevention*. Kuala Lumpur, Malaysia; 2013.
24. Gibb DM, Kizito H, Russell EC, et al. Pregnancy and infant outcomes among HIV-infected women taking long-term ART with and without tenofovir in the DART trial. *PLoS medicine* 2012; **9**(5): e1001217.
25. Committee APRS. Antiretroviral Pregnancy Registry International Interim Report for 1 January 1989 through 31 January 2013. In. Wilmington, NC: Registry Coordinating Center; 2013.
26. Moodley D, Esterhuizen T, Reddy L, et al. Incident HIV infection in pregnant and lactating women and its effect on mother-to-child transmission in South Africa. *The Journal of infectious diseases* 2011; **203**(9): 1231-4.
27. Effect of breastfeeding on infant and child mortality due to infectious diseases in less developed countries: a pooled analysis. *The Lancet* 2000; **355**(9202): 451-5.
28. Bahl R, Frost C, Kirkwood BR, et al. Infant feeding patterns and risks of death and hospitalization in the first half of infancy: multicentre cohort study. *Bulletin of the World Health Organization* 2005; **83**(6): 418-26.
29. Ahmed F, Clemens JD, Rao MR, Sack DA, Khan MR, Haque E. Community-based evaluation of the effect of breast-feeding on the risk of microbiologically confirmed or clinically presumptive shigellosis in Bangladeshi children. *Pediatrics* 1992; **90**(3): 406-11.
30. Lamberti LM, Fischer Walker CL, Noiman A, Victora C, Black RE. Breastfeeding and the risk for diarrhea morbidity and mortality. *BMC public health* 2011; **11 Suppl 3**: S15.
31. Victora CG, Smith PG, Vaughan JP, et al. Evidence for protection by breast-feeding against infant deaths from infectious diseases in Brazil. *Lancet* 1987; **2**(8554): 319-22.
32. Matthews LT, Sibeko S, Mansoor LE, Yende-Zuma N, Bangsberg DR, Karim QA. Women with Pregnancies Had Lower Adherence to 1% Tenofovir Vaginal Gel as HIV Preexposure Prophylaxis in CAPRISA 004, a Phase IIB Randomized-Controlled Trial. *PLoS ONE* 2013; **8**(3): e56400.
33. Flynn PM, Mirochnick M, Shapiro DE, et al. Pharmacokinetics and safety of single-dose tenofovir disoproxil fumarate and emtricitabine in HIV-1-infected pregnant women and their infants. *Antimicrobial agents and chemotherapy* 2011; **55**(12): 5914-22.
34. Shapiro RL, Holland DT, Capparelli E, et al. Antiretroviral concentrations in breast-feeding infants of women in Botswana receiving antiretroviral treatment. *The Journal of infectious diseases* 2005; **192**(5): 720-7.
35. Mirochnick M, Thomas T, Capparelli E, et al. Antiretroviral concentrations in breast-feeding infants of mothers receiving highly active antiretroviral therapy. *Antimicrobial agents and chemotherapy* 2009; **53**(3): 1170-6.
36. Van Rompay KK, Hamilton M, Kearney B, Bischofberger N. Pharmacokinetics of tenofovir in breast milk of lactating rhesus macaques. *Antimicrobial agents and chemotherapy* 2005; **49**(5): 2093-4.

37. Benaboud S, Pruvost A, Coffie PA, et al. Concentrations of tenofovir and emtricitabine in breast milk of HIV-1-infected women in Abidjan, Cote d'Ivoire, in the ANRS 12109 TEmAA Study, Step 2. *Antimicrobial agents and chemotherapy* 2011; **55**(3): 1315-7.
38. Line Alleslev Larsen SI, and Gideon Koren. Prediction of Milk/Plasma Concentration Ratio of Drugs. *The Annals of pharmacotherapy* 2003; **37**: 1299-306.
39. Mirochnick M, Taha T, Kreitchmann R, et al. Pharmacokinetics and Safety of Tenofovir in HIV-Infected Women During Labor and Their Infants During the First Week of Life. *JAIDS Journal of Acquired Immune Deficiency Syndromes* 2014; **65**(1): 33-41 10.1097/QAI.0b013e3182a921eb.
40. Gibb DM, Kizito H, Russell EC, et al. Pregnancy and Infant Outcomes among HIV-Infected Women Taking Long-Term ART with and without Tenofovir in the DART Trial. *PLoS Med* 2012; **9**(5): e1001217.
41. Beigi RH, Noguchi L, Brown G, Piper J, Watts DH. Performing Drug Safety Research During Pregnancy and Lactation: Biomedical HIV Prevention Research as a Template. *Journal of women's health (2002)* 2013.
42. Hendrix CW, Chen BA, Guddera V, et al. MTN-001: Randomized Pharmacokinetic Cross-Over Study Comparing Tenofovir Vaginal Gel and Oral Tablets in Vaginal Tissue and Other Compartments. *PLoS ONE* 2013; **8**(1): e55013.
43. Keller MJ, Madan RP, Torres NM, et al. A Randomized Trial to Assess Anti-HIV Activity in Female Genital Tract Secretions and Soluble Mucosal Immunity Following Application of 1% Tenofovir Gel. *PLoS ONE* 2011; **6**(1): e16475.
44. King T, Bushman L, Kiser J, et al. Liquid chromatography-tandem mass spectrometric determination of tenofovir-diphosphate in human peripheral blood mononuclear cells. *Journal of chromatography B, Analytical technologies in the biomedical and life sciences* 2006; **843**(2): 147-56.
45. Division of AIDS Table for Grading the Severity of Adult and Pediatric Adverse Events Version 1.0 December, 2004; Clarififcation August 2009.

Enrollment Consent

An open-label, short-duration, repeat-dose study of breast milk excretion and infant absorption of daily oral tenofovir disoproxil fumarate/emtricitabine when used by HIV-1 uninfected lactating women

Version 1.0
08 August 2014

INVESTIGATORS:

Investigator	Title	Institution	Telephone Contact
Nelly Rwamba Mugo	MBChB, MMed, MPH	Kenya Medical Research institute	0723914057
Kenneth Ngure	MPH, PhD	Jomo Kenyatta University of Agriculture and Technology	06722561
Elizabeth Irungu	MBChB, MPH	Thika Partners in Prevention Study, Kenyatta National Hospital	06722561
Njambi Njuguna	MBChB, MPH	Thika Partners in Prevention Study, Kenyatta National Hospital	06722561
Jared Baeten	MD, PhD	University of Washington, Seattle	2065203808
Kenneth Mugwanya	MD, MS	University of Washington, Seattle	2065203806

INFORMED CONSENT

We are asking you to volunteer to participate in this research study. The study is for breast feeding women without HIV and their infants. Before you decide if you want to join the study, we would like to explain the purpose, the risks and benefits, and what would be expected of you if you agree to be in the study.

If you agree to join the study, we will ask you to sign this consent form or make your mark in front of a witness. We will give you a copy of this form. This consent form might contain some words that are unfamiliar to you. Please ask us to explain anything you may not understand.

SOURCE OF FUNDING

This study is sponsored by the University of Washington and the Bill and Melinda Gates Foundation located in Seattle, WA, USA.

PURPOSE OF THE STUDY

Research studies have shown that taking a medication called Truvada® can lower the chances for HIV-**uninfected** men and women to get the HIV virus from their partner by ~75 percent. In those research studies, those people who took the Truvada® medication every day reduced their chances of getting HIV by as much as 90 percent. When HIV-uninfected people take medication to prevent getting the HIV virus it is called **pre-exposure prophylaxis (PrEP)**. Research studies have also shown that persons who take Truvada® medication rarely get serious side effects.

KEMRI / NERC
APPROVED
25 AUG 2014
P.O. Box 54840 00200, NAIROBI

Other research studies have found that pregnant women who take the Truvada® medication do not have increased risks of serious problems with their pregnancy and their babies do not have serious birth defects. More research studies in HIV-uninfected women who are taking Truvada® medication during pregnancy and breast feeding are needed.

The purpose of this study is to find out more about using Truvada® during breast feeding. Specifically, this research will study the amount of Truvada® drugs that are transferred into breast milk and then absorbed by the nursing baby when Truvada® is used by breast feeding women as PrEP.

YOUR PARTICIPATION IS VOLUNTARY

Your participation in this is voluntary. This consent form gives information about the study that we will discuss with you. Once you understand the study, and if you agree to take part, we will ask you to sign your name or make your mark on this form. We will offer you a copy to keep.

Before you learn about the study procedures, it is important that you know the following:

- You do not have to participate in the study if you do not want to.
- You may decide not to take part in this study, or to withdraw from the study at any time, without losing the benefits of your or your child's routine medical care and without losing the opportunity to participate in other studies.

If you decide to join the study, you and your baby will be in this study for up to 10 days.

ALTENATIVE TO PARTICIPATION IN THIS STUDY

You may decide not to take part in this, without losing the benefits of your routine medical care. If you decide not to join this study, you can still join another research study later, if one is available and you qualify.

STUDY PROCEDURES

If you decide to join the study, you will have daily study visits up to 10 visits.

At day 1 visit and each study visit you:

- Will be asked questions about your health and medical history, including whether you have been sick, your breastfeeding behaviors, and your feelings about taking medication for HIV prevention.
- Will talk with study staff about ways to avoid HIV and other infections passed during sex. We encourage you to have this counseling with your partner, but you can have it by yourself if you wish. You will be offered condoms.
- Will get medical care or referrals for medical care and other services if you need them.
- Will be asked to give updated information on where you live and how to keep in contact with you. The study staff will use this information to remind you of scheduled visits. If you don't return to the clinic on schedule, the study staff may try to contact you by phone or by visiting your home. They may try to reach you through the contact people that you list. If they talk to these people, they will not tell them why they are trying to reach you.

KEMRI / NERC APPROVED 25 AUG 2014 P.O. Box 54840-00200, NAIROBI

- Study staff will give you daily Truvada® tablet medication to take in their presence (called directly observed therapy). You will be counseled about possible drug side effects.

At your 7th and 10th visit:

- Study staff will give you the respective 7th and 10th directly observed therapy Truvada® medication dose to take.
- We will ask your permission to obtain two blood samples each [up to 10 mL / about 1 tablespoon at each sampling visit] at visit 7 and 10. The first blood sample will be collect right before you take the respective 7th and 10th doses of Truvada® medication. Then, the second blood sample will be collected between 1 and 2 hours after you have swallowed the respective 7th and 10th doses of Truvada® medication.
- We will ask you to obtain two breast milk samples [of up to 10 mL / about 1 tablespoon at each visit].The first breast milk sample will be collected right before you take your respective 7th and 10th doses of Truvada® medication. Then, the second breast milk sample will be collected within 1 to 2 hours after your respective 7th and 10th doses of Truvada® medication. Study staff will teach you how to obtain the breast milk samples.
- At visit 10 or last visit, you will also have your blood checked to assess the function of your kidneys.
- The blood and breast milk samples will be used by study researchers for tests to determine the amount of study drugs in the samples.

STUDY PROCEDURES FOR THE INFANTS

- If you decide to join the study, additional study procedures for the infant will only be required at visit day 1 and on your study visit 7. However, we encourage you to come with the baby at your daily study visits or if you feel the baby is not well. During each infant study visit, study staff will perform a physical examination of the baby to check how the baby is growing or if they have any problem. The infant's weight will be measured.
- At day 1 visit, we will obtain a small blood sample by a needle prick on the baby's finger or heel for an HIV antibody test. The study staff will talk with you about the HIV test and what it may mean to know the infant's HIV test results. Although it is very unlikely for the infant to have HIV infection if the mother is HIV uninfected, on very rare occasions it is possible for the infant to have HIV exposure from procedures like medical injections or blood transfusion, or through breastfeeding from a woman other than the mother. If the baby is found to be infected with HIV, study staff will counsel you on how to deal with it. We will refer you to an appropriate health facility for further check-up and no further study procedures will be performed on you and infant.
- At your study visit 7, we will do an examination of the baby to collect information on how much the baby is growing. We will ask your permission to obtain a blood sample from the baby [up to 2 mL / ½ teaspoon] at this visit. The blood sample will be used by study researchers for tests to determine the amount of the Truvada® medication in the infant's blood. If for any reason, infant blood sample cannot be collected on study visit 7, the sample can collected at any visit between visit 7 and visit 10 inclusive but before the end of the study.

KEMRI / NERC APPROVED 25 AUG 2014 P.O. Box 54840-00200, NAIROBI

At any time in the study:

- If the study staff thinks you or your baby may have any health problems, you may need to undergo a physical exam and may need to provide blood or other samples for testing.
- If you decide to leave the study before your last scheduled study visit, you have the right to refuse the final visit and not come again without giving any explanation.

USE OF SAMPLES AND DATA

We will store your data, blood, and breast milk samples only for testing in this study. Samples collected for testing drug levels will be shipped to the University of Washington and John Hopkins University in USA to conduct testing for drug levels. Any samples left over after completion of study specified testing will be destroyed. After testing for this study is completed, all samples will be destroyed. Before the infant and your samples leave the clinic, they will be assigned a code and your name will not be on them. Your name will be linked to the code only at this clinic and until December 31st, 2015 when the study data collection is expected to be completed. After that time, the link between your name and the code on your data and samples will be destroyed.

RISKS AND/OR DISCOMFORTS TO YOU

You may feel discomfort or pain when your blood is drawn. You may feel dizzy or faint. You may have a bruise where the needle goes into your arm.

You may become embarrassed, worried, or anxious when talking about your sexual practices, ways to protect against HIV and other infections passed during sex, and your HIV test results. You may become worried or anxious while waiting for your test results. If you become infected with HIV, knowing this could make you worried or anxious. Talking about HIV and finding out your test results could cause problems between you and your partner. You may also become worried or anxious when your child's blood is drawn or when the child cries. Trained counselors will help you deal with any feelings or questions you may have.

The study staff will make every effort to protect your privacy and confidentiality while you are having the study procedures. However, it is possible that others may learn of your participation here and, because of this, may treat you unfairly or discriminate against you. For example, you could have problems getting or keeping a job, or being accepted by your family or community.

RISKS AND/OR DISCOMFORTS TO INFANT

The infant may feel discomfort or pain when their blood is drawn. They may cry. They may have a bruise where the needle goes into their body at the site of blood draw. Trained counselors will help you and the infant deal with any feelings or questions you may have.

Risks potentially related to the Truvada® medication

You may have symptoms or adverse effects while participating in the study. These symptoms or adverse effects may be due to participation in the study or due to illnesses that have no relation to the study, like a cold or flu. All persons who participate in this study will be watched carefully to monitor their health. You should tell the doctor of the study clinic about any symptoms that you feel while you are participating in the study. You will be given a telephone number where

the study doctors will be available 24 hours a day, 7 days a week. You should call them if you experience any serious symptoms.

The adverse effects that can occur in a small proportion of people taking Truvada® are well known because the medication has been used by many people. Occasional adverse effects include: mild problems of kidney function that are only detected by laboratory tests; lack of energy/fatigue; upset stomach, vomiting, soft or liquid stools; dizziness. Rare adverse effects include: rash; liver function problems; serious kidney damage; allergic reaction. Small changes in the bones were observed in studies of people who were given Truvada®, but these changes did not cause any fractures, or other problems that bothered the patients. Change in the blood acid level (lactic acidosis) has occurred in some HIV-infected persons taking Truvada®, in combination with other drugs. Lactic acidosis can produce shortness of breath, nausea, and liver failure. This is a serious adverse effect of some medications used for HIV infection.

Truvada® has been studied in a small number of women during pregnancy and after birth and there are some studies that are still ongoing. Data from animals and people, including studies done in Kenya and Uganda, suggest that Truvada® is safe when used by women who are pregnant. Studies also suggest that Truvada® does not increase the risk of major birth defects for babies. However, not enough studies have been done in pregnant and breastfeeding women yet.

Risk of acquiring HIV infection and drug resistance

When a woman is breast feeding, she may have more risk of getting HIV than usual. You may become infected with HIV during this study from your partner or from other sexual partners you may have. It is very important to use all the known strategies to prevent getting HIV, like using condoms for all sexual relations and keeping your number of sexual partners low. You could become infected with a strain of HIV that could be resistant to Truvada® or other medicines used for HIV treatment. Resistance to antiretroviral medicines may make effective HIV treatment more difficult and may limit your treatment options. You will be able to discuss treatment and the generation of resistance to medicines with the study doctor.

For more information about risks of this study, ask your study doctor.

BENEFITS

You may get no direct benefit from being in this study. You or others may benefit in the future from information learned in this study. You also may get some personal satisfaction from being part of research on HIV.

You will be receiving Truvada®, which is known to help keep HIV-uninfected individuals from getting HIV. You will get counseling and testing for HIV. You will get free condoms. While Truvada® has been shown in research studies to lower the chances of getting HIV, it does not provide 100% protection; using condoms every time you have sex is important to protect against HIV. If you have health problems that may be due to infections passed during sex, you will get medicine to treat them, if needed. For other health problems, the study staff will give you care and treatment that is available at the clinic. For care and treatment that is not available at the clinic, study staff will tell you about other places where care and treatment may be available.

KEMRI / NERC
APPROVED
25 AUG 2014
P.O. Box 54840-00200, NAIROBI

After the study is over, the study staff will tell you organizations where you can seek HIV prevention services.

NEW FINDINGS

You will be told any new information learned during this study that is important for you or your baby's health or might cause you to change your mind about staying in the study. You will be told when the results of the study may be available, and how to learn about them.

COSTS TO YOU

There is no cost to you for being in this study. Treatments available to you from the study will be given free of charge.

REASONS WHY YOU MAY BE WITHDRAWN FROM THE STUDY

You may be removed from the study without your consent for the following reasons:

- The study is stopped or canceled.
- The researchers may determine that this study may not be a good fit for you or may not be safe for you or your baby

REIMBURSEMENT

You will receive Kshs. 400 at each scheduled visit for your time, effort and transport reimbursement.

CONFIDENTIALITY

Efforts will be made to keep your personal information confidential. However, absolute confidentiality cannot be guaranteed. Your personal information may be disclosed if required by law. Any sample from you or information about you will be identified only by code. The link between your name and code will be kept in a secure location at the clinic only. We will not discuss any information about you with your partner unless you give written permission, and we will encourage you to be present during the discussion. Any publication of this study will not use your name or identify you personally. Government or university staff sometimes review studies such as this one to make sure they are being done safely and legally. If a review of this study takes place, your records may be examined. The reviewers will protect your privacy. The study records will not be used to put you at legal risk of harm. The U.S. Food and Drug Administration (FDA) reserve the right to review study data that may contain identifying information.

Your study records may be reviewed by study staff and representatives of:

- University of Washington
- Bill and Melinda Gates Foundation
- Pharmacy and Poisons Board
- Kenya Medical Research Institute
- National Council of Science and Technology (NACOSTI).

RESEARCH-RELATED INJURY

The study staff will monitor your health while you are in this study. If you or your baby has any health problems between visits, please contact the study staff. If you have a medical emergency

KEMRI / NERC APPROVED 25 AUG 2014 P. O. Box 54840-00200, NAIROBI

that requires immediate care, please visit the nearest health facility and call Dr. Elizabeth Irungu at the Thika Research Clinic on 0736464299.

If you or your baby are injured as a result of participating in this study, you will be offered care at the study clinic, free of charge. It is important that you tell the members of the team of researchers at this clinic if you feel that you have been injured because of taking part in this study. If you require medical care that the study clinic cannot provide, the study doctors will refer you to the appropriate services or organizations that can provide care for the injury or the University of Washington will reimburse you for treatment of injury or illness resulting from the study according to University of Washington policy. You do not give up any legal rights by signing this consent form.

PROBLEMS OR QUESTIONS

If you ever have any questions about the study, you should contact Dr. Kenneth Ngure at the Thika Partners in Prevention Study clinic at Tel. 067-21305/22561.

If you have questions about your rights as a research participant, you should contact the Secretary of the KEMRI Ethics Review Committee P. O. Box 54840-00200, Nairobi: Telephone number: 020-272-2541, 0722-205901, 0733-400003.Email address: ERCadmin@kemri.org

STATEMENT OF CONSENT AND SIGNATURES

I have read this form or had it read to me. I have discussed the information with study staff. My questions have been answered. I understand that my decision whether or not to take part in the study is voluntary. I understand that if I decide to join the study I and the infant may withdraw at any time. By signing this form I and the infant do not give up any rights that we have as a research participants.

____________________	____________________	__________
Participant Name (Print)	Participant Signature/Thumbprint	Date
____________________	____________________	__________
Study Staff Conducting Consent Discussion (print)	Study Staff Signature	Date
____________________	____________________	__________
Witness Name (Print)	Witness Signature	Date

KEMRI / NERC APPROVED 25 AUG 2014 P.O. Box 54840-00200, NAIROBI

FATHER'S STATEMENT OF PERMISSION FOR THE INFANT TO JOIN THE STUDY

We are asking you for permission to allow your child participate in this research study. The study is for breast feeding women without HIV and their babies. Before you decide if you want your child to join the study, we would like to explain the purpose, the risks and benefits, and what would be expected of the baby if you agree to your child to be in the study.

If you agree to the child to join this study, we will ask you to sign this permission form or make your mark. We will give you a copy of this form. This permission form might contain some words that are unfamiliar to you. Please ask us to explain anything you may not understand.

PURPOSE OF THE STUDY

Research studies have shown that taking a medication called Truvada® can lower the chances for HIV-uninfected men and women to get the HIV virus from their partner by ~75 percent. In those research studies, those people who took the Truvada® medication every day reduced their chances of getting HIV by as much as 90 percent. When HIV-uninfected people take medication to prevent getting the HIV virus it is called **pre-exposure prophylaxis (PrEP)**. Research studies have also learned that Truvada® is safe and does not lead to significant health problems in persons who take the medication.

Other research studies have found that pregnant women who take the Truvada® medication do not have increased risks of serious problems with their pregnancy and their babies do not have serious birth defects. More research studies in HIV-uninfected women who are taking Truvada® medication during pregnancy and breast feeding are needed.

The purpose of this study is to find out more about using Truvada® during breast feeding. Specifically, this research will study the amount of Truvada® drugs that are transferred into breast milk and then absorbed by the nursing baby when Truvada® is used by breast feeding women as PrEP

INFANT STUDY PROCEDURES

If both you and the mother agree that the infant join the study, the mother and the infant will be in this study for up to 10 days. The baby will be required to attend at least two study visits, including day 1 visit and the mother's study visit 7. However, we encourage the mother to come with the baby at her daily study visits or if you feel the baby is not well. During each infant study visit, study staff will perform a physical examination of the baby to check on how the baby is growing or if they have any problem.

At the mother day 1 visit, we will obtain a small blood sample by a needle prick on the baby's finger or heel for an HIV antibody test. Although it is very unlikely for the infant to have HIV infection if the mother is HIV uninfected, on very rare occasions it is possible for the infant to have HIV exposure procedures like through medical injections or blood transfusion, or breastfeeding from a woman other than the mother. If the baby is found to be infected with HIV, study staff will counsel you and the mother on how to deal with it. We will refer the baby to the appropriate health facility for further check-up and both the mother and the infant will have no further study procedures.

KEMRI / NERC APPROVED 25 AUG 2014 P.O. Box 54840-00200, NAIROBI

At the mother's 7th visit, the infant;

- Will have a clinical assessment to determine if they are in good health. They will have their weight taken.
- Will have a single blood sample [maximum of 2 mL / about ½ teaspoon] collected after the mother's 7th Truvada® medication dose. If for any reason, infant blood sample cannot be collected on stud visit 7, the sample will be collected at any visit between visit day 7 and visit 10 inclusive but before the end of the study.

RISKS AND/OR DISCOMFORTS TO INFANT

The baby may feel discomfort or pain when their blood is drawn. They may cry. They may have a bruise where the needle goes into their body at the site of blood draw. Trained staff will help the mother and the infant to deal with any feelings or questions the mother may have.

BENEFITS

There are no direct benefits to infant from being in this study. The infant or others may benefit in the future from information learned in this study.

PROBLEMS OR QUESTIONS

If you ever have any questions about the study, you should contact Dr. Kenneth Ngure at the Thika Partners in Prevention Study clinic at Tel. 067-21305/22561.

If you have questions about your rights as a research participant, you should contact the Secretary of the KEMRI Ethics Review Committee P. O. Box 54840-00200, Nairobi: Telephone number: 020-272-2541, 0722-205901, 0733-400003.Email address: ERCadmin@kemri.org

STATEMENT OF PERMISSION AND SIGNATURES

I have read this form or had it read to me. I have discussed the information with study staff. My questions have been answered. I understand that my decision whether or not to let my child take part in the study is voluntary. I understand that if I agree to the child to join the study the child may withdraw at any time. By signing this form my child does not give up any rights that they have as a research participant.

__________	__________	__________
Participant Name (Print)	Participant Signature/Thumbprint	Date
__________	__________	__________
Study Staff Conducting Study Permission Discussion (print)	Staff Signature	Date
__________	__________	__________
Witness Name (Print)	Witness Signature	Date

KEMRI / NERC
APPROVED
25 AUG 2014
P. O. Box 54840-00200, NAIROBI

FATHER NOT REASONABLY AVAILABLE

Mother's Name (Print)	Mother's Signature/Thumbprint	Date
Study Staff Conducting Study	Staff Signature	Date

Copies to: 1. Investigators

2. Study Participant

KEMRI / NERC APPROVED 25 AUG 2014 P.O. Box 54840-00200, NAIROBI
